# Heterochromatin delays CRISPR-Cas9 mutagenesis but does not influence the outcome of mutagenic DNA repair

**DOI:** 10.1371/journal.pbio.2005595

**Published:** 2018-12-12

**Authors:** Eirini M. Kallimasioti-Pazi, Keerthi Thelakkad Chathoth, Gillian C. Taylor, Alison Meynert, Tracy Ballinger, Martijn J. E. Kelder, Sébastien Lalevée, Ildem Sanli, Robert Feil, Andrew J. Wood

**Affiliations:** 1 MRC Human Genetics Unit, Institute of Genetics and Molecular Medicine, University of Edinburgh, Edinburgh, United Kingdom; 2 Institute of Molecular Genetics of Montpellier (IGMM), CNRS and University of Montpellier, Montpellier, France; National Cancer Institute, United States of America

## Abstract

Genome editing occurs in the context of chromatin, which is heterogeneous in structure and function across the genome. Chromatin heterogeneity is thought to affect genome editing efficiency, but this has been challenging to quantify due to the presence of confounding variables. Here, we develop a method that exploits the allele-specific chromatin status of imprinted genes in order to address this problem in cycling mouse embryonic stem cells (mESCs). Because maternal and paternal alleles of imprinted genes have identical DNA sequence and are situated in the same nucleus, allele-specific differences in the frequency and spectrum of mutations induced by CRISPR-Cas9 can be unequivocally attributed to epigenetic mechanisms. We found that heterochromatin can impede mutagenesis, but to a degree that depends on other key experimental parameters. Mutagenesis was impeded by up to 7-fold when Cas9 exposure was brief and when intracellular Cas9 expression was low. In contrast, the outcome of mutagenic DNA repair was unaffected by chromatin state, with similar efficiencies of homology-directed repair (HDR) and deletion spectra on maternal and paternal chromosomes. Combined, our data show that heterochromatin imposes a permeable barrier that influences the kinetics, but not the endpoint, of CRISPR-Cas9 genome editing and suggest that therapeutic applications involving low-level Cas9 exposure will be particularly affected by chromatin status.

## Author summary

Genomic imprinting is a developmental process through which either the maternally or paternally derived allele of a gene is epigenetically silenced. Imprinting has served as a model system to understand the mechanisms through which chromatin modifications can influence transcriptional regulation; comparisons between active and repressed alleles in the same cell nucleus provide an internal control for the effects of DNA sequence properties and exposure to *trans*-regulators (elements that modify distant genes). Here, we have exploited these features in order to understand how chromatin modifications influence genome editing with the clustered regularly interspaced short palindromic repeats (CRISPR)-CRISPR-associated protein 9 (Cas9) system. By comparing the frequency and spectrum of Cas9-induced genome edits on maternal versus paternal alleles of imprinted genes, we discovered experimental conditions that determine whether chromatin modifications have a large or small effect on the outcome of genome editing procedures. We show that chromatin state does not affect the size of deletions and insertions or their relative frequency compared to precise genome edits specified by an oligonucleotide donor template. However, repressed chromatin inhibits the rate at which mutations form, particularly when the intracellular concentration of Cas9 is low. At longer exposure times, mutation frequencies on repressed versus active alleles are similar. Collectively, our data suggest that chromatin status affects the kinetics but not the endpoint of genome editing experiments.

## Introduction

Clustered regularly interspaced short palindromic repeats (CRISPR)-CRISPR-associated protein 9 (Cas9) is an RNA-guided endonuclease involved in bacterial adaptive immunity, which has been repurposed as a highly efficient tool for eukaryotic genome editing [1–3]. In its natural form, Cas9 protein associates with a duplex of two RNA molecules: the CRISPR RNA (crRNA), which recognises a short section of target DNA (the ‘protospacer’), and a *trans*-activating crRNA, which acts as a scaffold to link the crRNA and Cas9 endonuclease. Most genome editing applications use a single guide RNA molecule (sgRNA) resulting from an engineered fusion of these two components. After target DNA cleavage, mutations arise through the action of cellular DNA repair pathways. Nonhomologous end joining (NHEJ; including both classical and microhomology-mediated pathways) can yield short insertions and deletions (InDels) suitable for gene knockout, whereas homology-directed repair (HDR) pathways utilise exogenous donor templates to introduce precise sequence changes.

It is well established that genetic properties of the genomic target site and sgRNA molecule have a significant effect on the efficiency of CRISPR mutagenesis [4–6]. However, Cas9, being prokaryotic in origin, did not evolve to cope with the complex chromatinised environment of the eukaryotic genome. Despite prior studies in this area [4,7–15], the extent to which epigenetic properties of the target site—including DNA and histone modifications—influence mutation frequency and DNA repair outcome remains incompletely understood. Stably positioned nucleosomes act as a barrier to Cas9 binding and function on synthetic chromatin fibres [7,8,11], and in vivo [7,15], yet catalytically dead Cas9 (dCas9) can open previously inaccessible regions of chromatin [16,17]. It has been reported that some sgRNAs show reduced activity within heterochromatin whereas others do not [13,14]. The reasons behind this paradox are unclear but presumably involve other experimental variables that modify the influence of chromatin on CRISPR activity. Furthermore, it is widely accepted that double strand break (DSB) repair is influenced by the chromatin environment in which DSBs arise [18–22], and DSB repair is central to the mechanism of genome editing [23,24]. However, it is unclear whether preexisting epigenetic properties of the target site impact upon the specific sequence changes that arise following Cas9 cleavage.

Genomic imprinting is a natural epigenetic process in which either the maternal or paternally derived copy of a gene is transcriptionally silenced. Essential regulatory elements within imprinted domains called ‘imprinting control regions’ undergo differential methylation at CpG dinucleotides during male and female gametogenesis. This leads to the establishment of monoallelic domains of heterochromatin in the early embryo that are maintained throughout somatic development [25]. These imprinted alleles carry all known hallmarks of constitutive heterochromatin, including post-translational histone modifications (H3K9me3, H4K20me3, histone hypoacetylation) and heterochromatin binding proteins (HP1γ) [26].

Genomic imprinting has provided numerous insights into mechanisms of transcriptional regulation [27–30]. Because active and silent alleles of imprinted loci have an identical DNA sequence, chromosomal position, and potential exposure to diffusible regulators, allele-specific chromatin modifications must be sufficient to account for their allele-specific expression [31]. Based on this principle, we postulated that genomic imprinting could be used to provide new insights into the influence of chromatin modifications on targeted mutagenesis.

## Results

**Fig 1:**
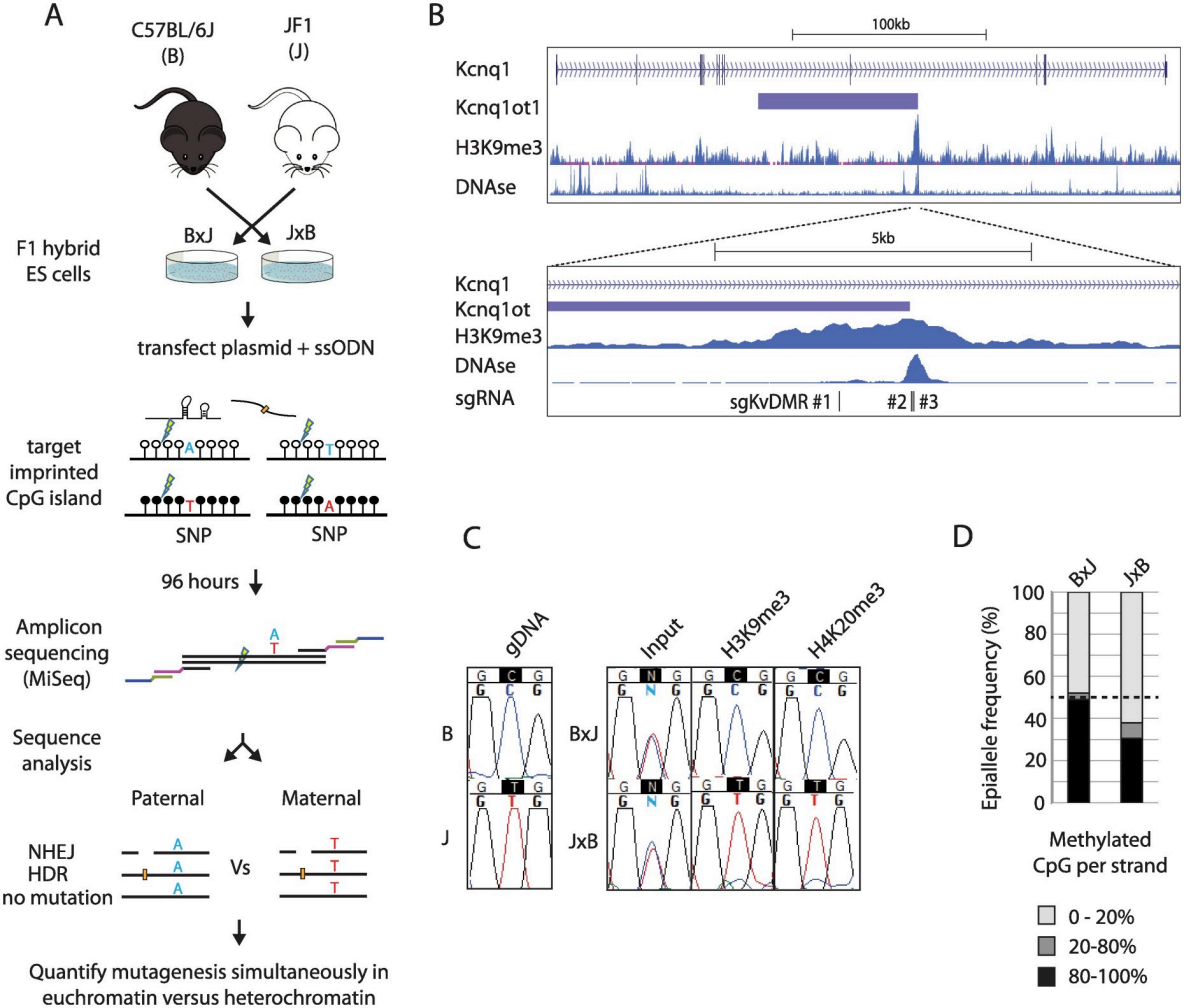
Imprinted chromatin as a model system to quantify epigenetic influences on genome editing. (A) Schematic outlining the experimental workflow. Throughout the text, F1 hybrid cell lines are depicted with the maternal strain denoted before the paternal strain (i.e., In B×J: B is maternal and J paternal). sgRNAs are designed to cleave approximately 40–100 bp from a heterozygous SNP within imprinted chromatin (open and closed circles). MiSeq amplicons span both the SNP and site of mutation, which allows simultaneous assessment of genome editing outcome and parental allele at high-throughput. (B) Top: schematic showing the imprinted mouse *Kcnq1* gene including H3K9me3 ChIP and DNase-I–seq data from mESCs available through EncODE (ENCSR000CFZ, GSM1014187) (bottom). Higher-resolution view of the *KvDMR* imprinted CpG island within *Kcnq1*, showing the position of three sgRNAs used in panel E. (C) Allele-specific enrichment of H3K9me3 and H4K20me3. PCR fragments spanning the target sites of sgKvDMR#2 and #3 were amplified from input, or ChIP DNA prior to Sanger sequencing across an allelic SNP. gDNA = genomic DNA from purebred mice. (D) Example of CpG methylation data from the *KvDMR* locus. Bisulphite-converted gDNA was subjected to Illumina amplicon sequencing across a region spanning 13 CpG dinucleotides (S1A Fig), and reads were classified according to the proportion of nonconverted (methylated) CpGs. The black dashed line indicates the expected level of methylation across all alleles when imprinting is completely maintained (50%). In subsequent editing experiments, the percentage of hypermethylated (>80%) strands is reported together with histograms showing allele-specific mutation frequency. Quantitative data underlying panel D are provided in S1 Data, and details of MiSeq libraries including SRA accessions are provided in S2 Data. ChIP, chromatin immunoprecipitation; gDNA, genomic DNA; HDR, homology-directed repair; mESC, mouse embryonic stem cell, NHEJ, nonhomologous end joining; sgRNA, single guide RNA; SNP, single nucleotide polymorphism; SRA, Sequence Read Archive; ssODN, single-stranded oligodeoxynucleotide.

Mouse embryonic stem cell (mESC) lines were derived from male F1 hybrid blastocysts of inter-subspecies crosses between (C57BL6/J [B6]) and the *Mus musculus molossinus* inbred strain JF1 (Fig 1A). These cells are heterozygous for strain-specific single nucleotide polymorphisms (SNPs) [32], which serve as genetic markers that distinguish maternal and paternal chromosomes. To control for possible genetic effects on mutagenesis arising from SNPs, we derived mESCs from reciprocal crosses (B6 female × JF1 male [B×J], and JF1 female × B6 male [J×B]) and used both cell lines in parallel wherever possible. Although mESCs show globally reduced CpG methylation compared to primary somatic cells, the effects on imprinted regions were minimised by using male (XY) cells cultured in serum-free conditions. Unless otherwise stated, cells were cultured in the presence of pharmacological inhibitors of GSK3β but not MEK signalling [33,34].

We targeted three maternally imprinted CpG islands: *KvDMR1* (hereafter referred to as *KvDMR*, Fig 1B, S1A Fig), *Impact* (S2A Fig), and *Inpp5f_v2* (S3A Fig). To determine whether these loci had distinct epigenetic configurations on maternal and paternal alleles in B×J and J×B mESCs, we performed allele-specific DNase-I hypersensitivity assays (S1B, S2B and S3B Figs) as well as allele-specific chromatin immunoprecipitation (ChIP) experiments for H3K9me3 and H4K20me3 (Fig 1C, S1C, S1D, S2C, S2D, S3C and S3D Figs). In each case, paternally derived alleles were substantially more sensitive to DNase-I digestion, whereas maternal alleles were highly enriched for heterochromatin marks. Nonetheless, in some cases, loss of imprinting (LOI) was evident from incomplete allelic enrichment of histone modifications (S3D Fig) and incomplete depletion of paternal alleles by DNase-I (S3B Fig). During subsequent editing experiments, we therefore quantified CpG methylation levels as a biomarker of LOI at the target site in mock-transfected cells (Fig 1D).

We designed three different sgRNAs to target protospacer sequences within KvDMR (Fig 1B, S1A Fig). mESCs were transfected with Cas9 and individual sgRNAs expressed from plasmid pX459v2 [35], together with a single-stranded oligodeoxynucleotide (ssODN) donor template that introduced point mutations to prevent re-cutting following HDR. Transfected cells were selected in puromycin and collected as a pool 96 hours after transfection. Editing was quantified by Illumina sequencing of PCR amplicons spanning both the site of cleavage and an allelic SNP (Fig 1A and S1A Fig, for detailed experimental protocols see Materials and methods). This allowed the outcome of mutagenic repair to be determined, at the nucleotide level, separately on maternal and paternal chromosomes.

**Fig 2:**
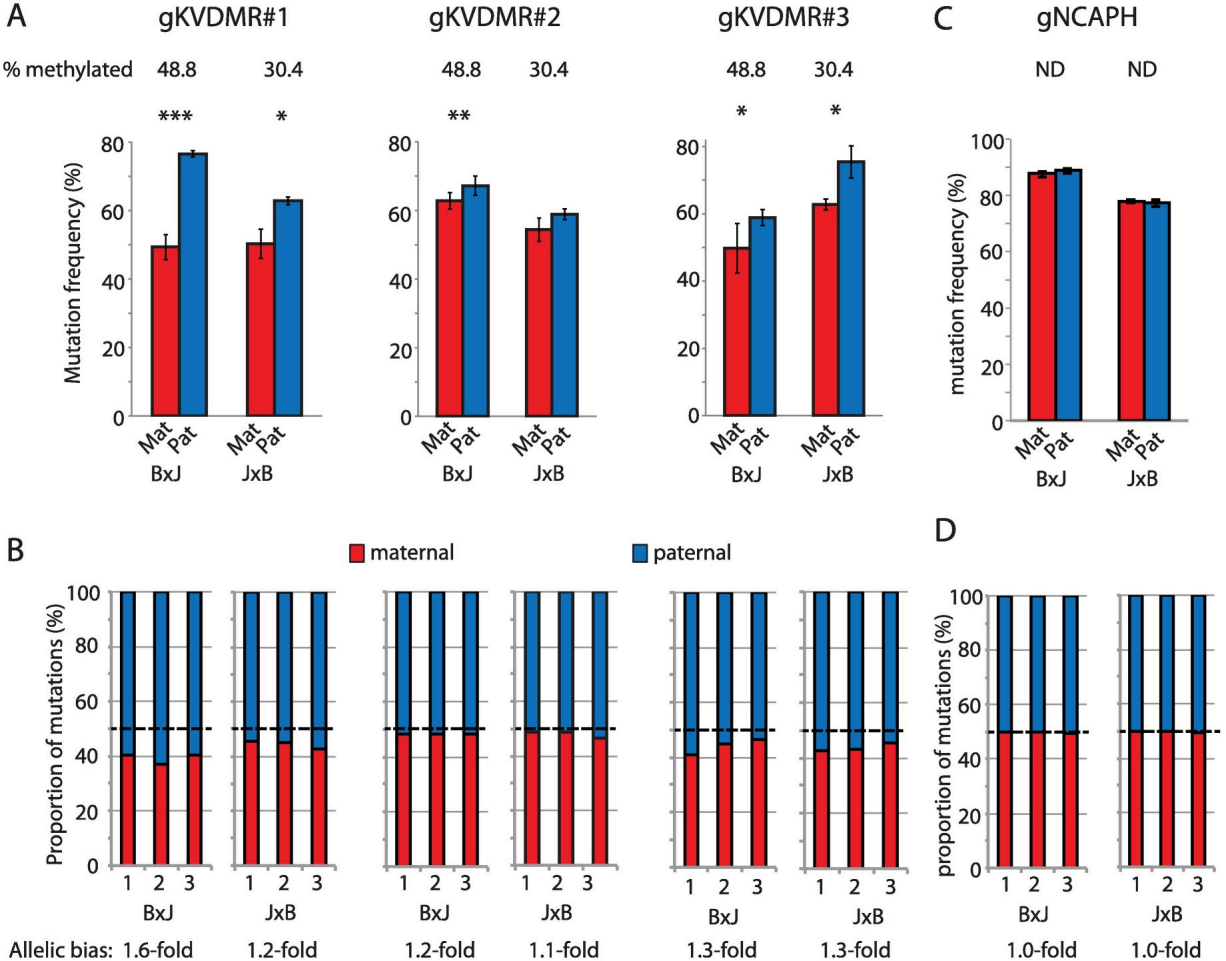
Lower mutation frequency on silenced alleles following 96-hour exposure. (A) Allele-specific mutation frequencies for KvDMR sgRNAs #1–3 in cells harvested 96 hours post transfection, following selection of transfectants in puromycin. Error bars represent SD of three biological replicates; *p*-values denote two-tailed paired *t* tests of difference between maternal and paternal alleles. *p < 0.05, **p < 0.01, ***p < 0.001. In this, and subsequent figures, the percentage of hypermethylated alleles in mock-transfected cells is shown above the histogram to indicate the degree of imprinting at the time of editing. (B) Stacked histograms show the allelic mutation bias in each experimental replicate. (C, D) Allele-specific mutation frequencies from experiments using an sgRNA (sgNCAPH) targeting a nonimprinted locus, presented as described in panels A and B. Quantitative data underlying all panels are provided in S1 Data, and details of MiSeq libraries including SRA accessions are provided in S2 Data. Mat, maternal; ND, methylation analysis not done; Pat, paternal; sgRNA, single guide RNA; SRA, Sequence Read Archive.

We first compared the frequency of all edits, including InDels and point mutations introduced from ssODN donors, on maternal versus paternal alleles. All three sgRNAs yielded more mutations on the active paternal allele compared to the repressed maternal allele (Fig 2A and 2B), whereas a control, nonimprinted locus (*NCAPH*) showed no such allelic bias (Fig 2C and 2D). The effect of imprinted chromatin was subtle in this context: 1.1- to 1.6-fold (Fig 2A and 2B), even in B×J cells in which imprinting was completely maintained (Fig 1D).

**Fig 3:**
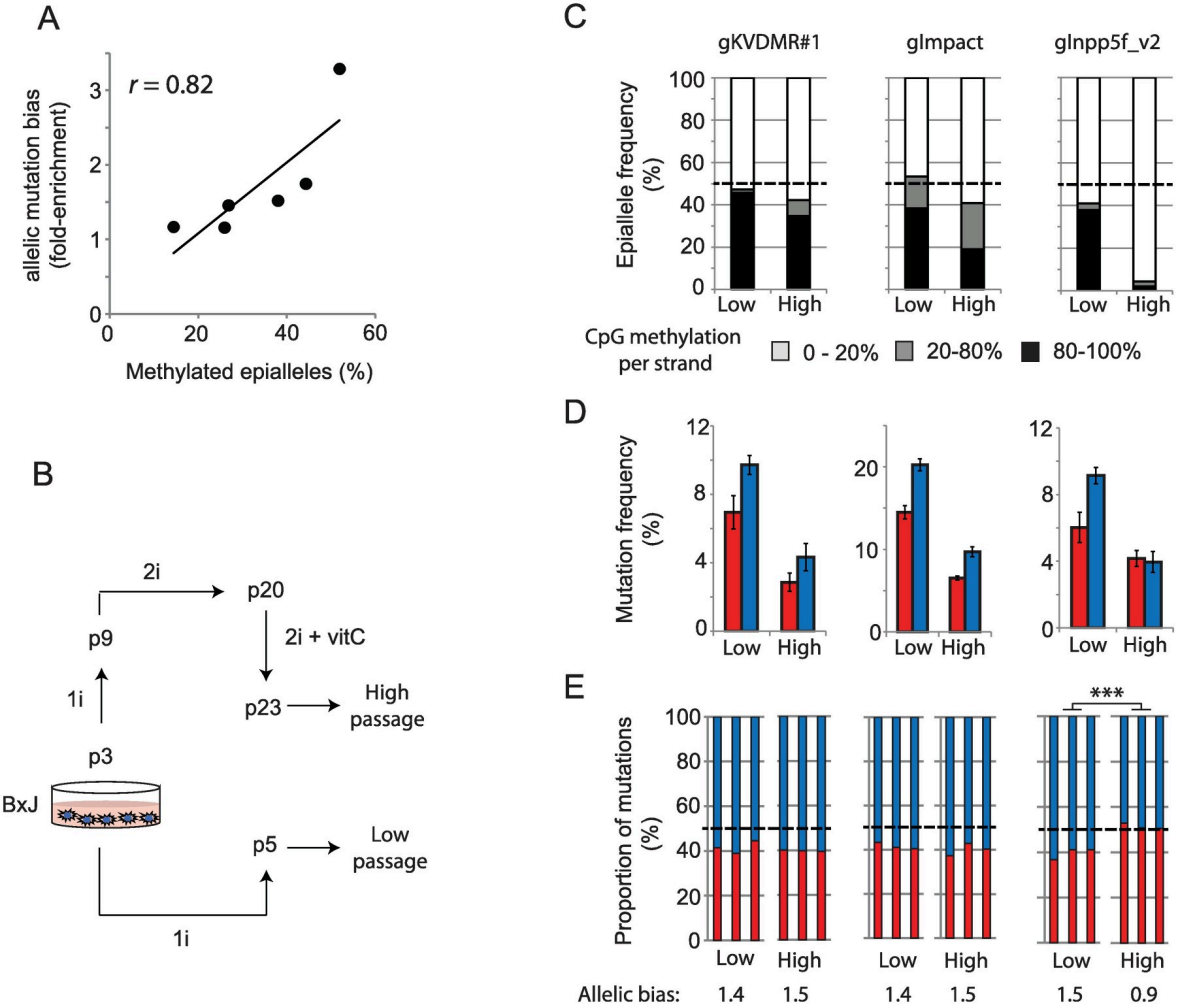
Allele-specific mutation bias is caused by allele-specific chromatin modifications. (A) The degree of methylated alleles correlates with allele-specific mutation bias across three imprinted target sites (sgInpp5f*_v2*, sgKvDMR1, sgImpact) in two mESC lines. Each point represents the mean enrichment of mutations on paternal alleles over biological triplicate editing experiments (y-axis) and a single CpG methylation measurement from mock-transfected cells (x-axis). (B) Schematic illustrating the derivation of high-passage cells in order to induce LOI. Media composition (1i, 2i) and cell culture protocols are detailed in Materials and methods. (C) CpG methylation levels at three imprinted loci in low- and high-passage mESCs (B×J only) following mock transfection. Note that only the *Inppf5_v2* locus has undergone extensive demethylation during culture. (D) Allele-specific mutation frequencies in low- versus high-passage cells. Error bars represent SD (*n* = 3) Transfectants were not selected in these experiments, which accounts for the lower overall mutation frequency relative to Fig 2. Transfection was also less efficient in high-passage cells. (E) Stacked histograms show the allelic mutation bias in each experiment. Asterisks denote *p*-values for unpaired *t* tests on the fold-difference between mutation frequencies on maternal and paternal alleles in low- compared to high-passage cells. ***p < 0.001. Quantitative data underlying panels A, C, D, and E are provided in S1 Data, and details of MiSeq libraries including SRA accessions are provided in S2 Data. LOI, loss of imprinting; mESC, mouse embryonic stem cell; SRA, Sequence Read Archive.

To ensure that the observed bias in allele-specific mutation frequency could be attributed to chromatin modifications, we took advantage of the stochastic LOI reported to occur in mESCs [33,34,36]. In a series of six triplicate mutagenesis experiments targeting three imprinted loci (sgKVDMR1, sgImpact, sg*Inpp5f_v2*) in two mESC lines, we observed a significant correlation between the degree of imprinted CpG methylation in mock-transfected cells and the degree of allele-specific mutation bias (r = 0.82, *p* < 0.05, Fig 3A). To experimentally induce LOI, a B×J cell line was cultured to high passage (p23) in the presence of MEK inhibitors (‘2i’) and vitamin C, which have been reported to induce global hypomethylation in mESCs [33,34,37]. CpG methylation was unaffected at the *KvDMR* locus, slightly reduced at *Impact*, and completely lost at *Inpp5f_v2* in late passage compared to early passage cells (Fig 3C). Accordingly, allele-specific mutation bias was lost at *Inpp5f_v2* but not at the *KvDMR* or *Impact* target sites (Fig 3D and 3E). This shows that chromatin modifications are sufficient to influence the frequency of mutations induced by CRISPR-Cas9 on identical target sites in the same cell nucleus, but the effects are typically subtle (<2-fold) in cells harvested 96 hours following plasmid transfection.

**Fig 4:**
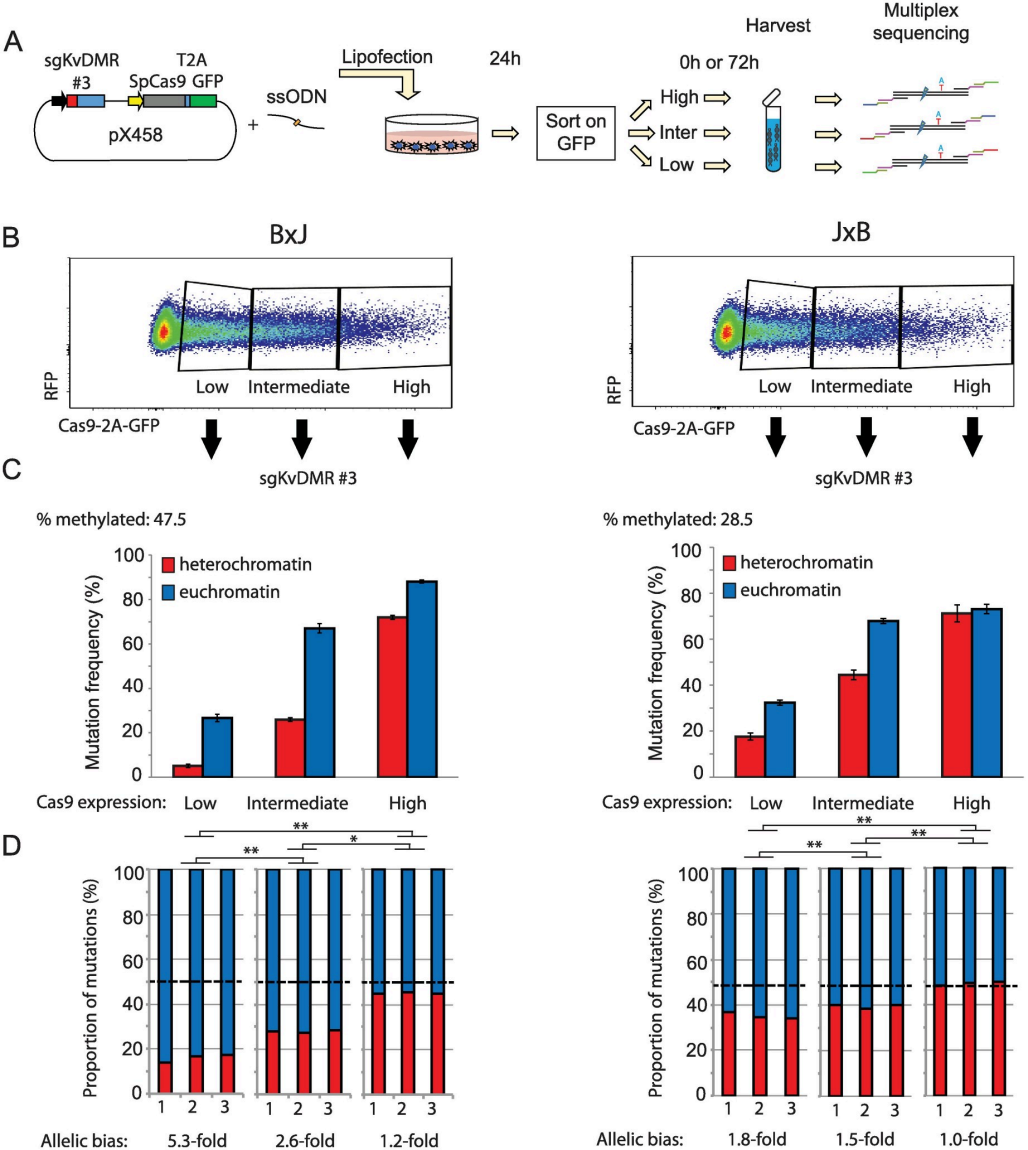
Heterochromatin impedes genome editing in a Cas9-concentration–dependent manner. (A) Schematic outlining the experimental workflow. After FACS, cells were either harvested immediately (S4 Fig) or cultured for an additional 72 hours (this figure). (B) Flow cytometry profiles show widely variable expression of Cas9-2A-eGFP at 24 hours following transfection with guide gKvDMR#3 expressed from pX458 (see panel A). (C) Allele-specific mutation analysis within cell populations expressing different levels of Cas9, FACS purified 24 hours post transfection using the gating scheme in panel B and then cultured for an additional 72 hours before harvesting. Allelic differences are less pronounced in J×B cells due to partial loss of imprinted heterochromatin on maternal alleles in this cell line. Error bars represent SD of three biological replicates. (D) Stacked histograms show the allelic mutation bias in each experiment. The percentage of methylated strands was measured in mock-transfected cells without selection for Cas9 expression level. One-way ANOVA was conducted using fold-difference between mutation frequencies on maternal versus paternal alleles, to assess whether this was affected by Cas9 expression level. Significant differences were found in both cell lines (*p* < 0.001). Asterisks denote *p*-values for Tukey’s HSD test on the specified pairwise comparisons. *p < 0.05, **p < 0.01. Quantitative data underlying panels C and D are provided in S1 Data, and details of MiSeq libraries including SRA accessions are provided in S2 Data. Cas9, CRISPR-associated protein 9; eGFP, enhanced green fluorescent protein; FACS, fluorescence-activated cell sorting; GFP, green fluorescent protein; HSD, honest significant difference; RFP, red fluorescent protein; SRA, Sequence Read Archive; ssODN, single-stranded oligodeoxynucleotide.

We next considered whether common experimental variables affect the degree to which modified chromatin impedes mutagenesis, reasoning that CRISPR might less efficiently overcome the heterochromatin barrier when the intracellular concentration of Cas9 is low [38]. To test this hypothesis, KvDMR sgRNA#3 was expressed from plasmid pX458, in which Cas9 is fused to enhanced green fluorescent protein (eGFP) via a self-cleaving 2A peptide. eGFP levels therefore serve as a reporter of Cas9 translation (Fig 4A). Flow cytometry revealed that Cas9 translation levels were highly variable between cells at 24 hours post transfection (Fig 4B). Cells were purified by fluorescence-activated cell sorting (FACS) into three categories based on eGFP fluorescence and then collected either immediately (24 hours, S4 Fig) or following an additional 3 days in culture (Fig 4). B×J cells expressing Cas9 at low levels showed a profound (5.3-fold) reduction in mutation frequency on the silent maternal compared to the active paternal allele after 4 days of exposure (Fig 4C). At intermediate levels of Cas9-eGFP expression, the mutational bias was moderate (2.6-fold), whereas high expression yielded only subtle differences between alleles (approximately 1.2-fold) (Fig 4C). J×B cells showed the same trend, but mutations on the maternal allele were more frequent, consistent with approximately 30% LOI in this cell line (Fig 4C). Heterochromatin therefore impedes mutagenesis to a greater extent when the intracellular concentration of Cas9 is low.

**Fig 5:**
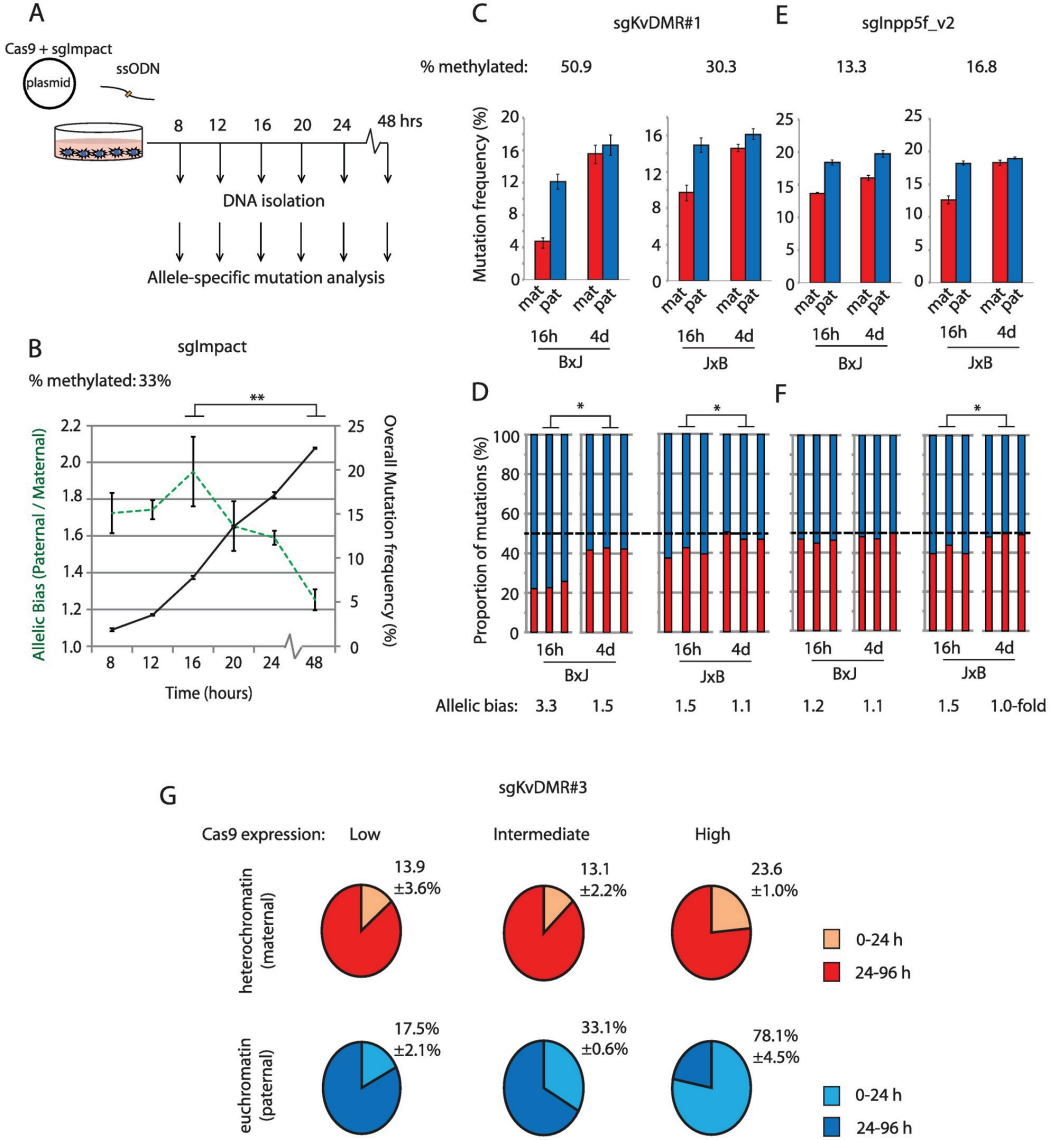
Heterochromatin impairs the kinetics of CRISPR mutagenesis. Schematic depicting the experimental workflow for the time course experiment in panel B. (B) Overlaid line graphs depict total mutation rates (black solid line, right y-axis) and fold-enrichment for mutations on the euchromatic paternal allele (green dashed line, left y-axis) over time using sgImpact targeting the imprinted *Impact* locus (S2 Fig) in B×J cells. Error bars represent SD of three biological replicates. Transfected cells were not selected in this experiment. (C, E) Allele-specific mutation frequencies at 16 and 96 hours post transfection for experiments using an sgRNA targeting KvDMR (sgKvDMR#1; S1A Fig). Error bars represent SD (*n* = 3). (D, F) Stacked histograms show the allelic mutation bias in each experiment. Asterisks denote *p*-values for unpaired *t* tests on the fold-difference between maternal and paternal allele mutation frequencies following brief (16-hour) compared to long (4-day) exposure high-passage cells. ***p < 0.001. (G) Pie charts show mutation frequencies observed 24 hours post transfection, expressed as a percentage of the mutation frequency in cells collected after 96 hours. Data are derived from the experiment described in Fig 4 and S4 Fig, with mutation frequencies broken down by both parental allele and Cas9 expression level. Experiments used sgKvDMR#3 in B×J cells collected either immediately after sorting on Cas9-2A-eGFP (24 hours) or after an additional 72 hours in culture. CIs indicate SD (*n* = 3 biological replicate transfections). Quantitative data underlying all panels are provided in S1 Data, and details of MiSeq libraries including SRA accessions are provided in S2 Data. Cas9, CRISPR-associated protein 9; eGFP, enhanced green fluorescent protein; sgRNA, single guide RNA; SRA, Sequence Read Archive; ssODN, single-stranded oligodeoxynucleotide.

Single particle tracking experiments have shown that the efficiency of target searching by dCas9 is reduced within heterochromatin [9]. Whether this impacts upon mutagenesis with Cas9 nuclease was not tested. To determine whether heterochromatin delays mutation kinetics, we initially targeted the *Impact* imprinted locus (S2 Fig), using a highly active sgRNA (sgImpact) that yielded similar frequencies of mutation on maternal and paternal alleles after 96 hours of exposure (S2F Fig). B×J cells were collected at 4-hour intervals following transfection, after which allele-specific mutagenesis was quantified as described above (Fig 5A). As expected, the frequency of mutations across both alleles increased steadily from 8 hours to 48 hours following transfection, but mutations were more skewed towards the active paternal allele at earlier compared to later time points (Fig 5B). Using sgRNAs targeting two additional imprinted loci (sgKvDMR#1 [S1 Fig] and sgInpp5f_v2 [S3 Fig]), we observed stronger skewing towards allelic target sites within euchromatin at early (16-hour) compared to later (96-hour) time points (Fig 5C–5F). This effect was most striking in cells exposed to high concentrations of Cas9, for which a large majority (78%) of mutations present in euchromatin following 96 hours of exposure were found to occur within the first 24 hours (Fig 5G, S4 Fig). Within heterochromatin, only 23% of mutations present at 96 hours had occurred by this earlier time point (Fig 5G, S4 Fig).

Allele-specific ChIP experiments were conducted to quantify dCas9 occupancy after targeting to the *Impact* and *KvDMR* imprinted loci (S5A Fig). This revealed an approximately 2-fold enrichment on euchromatic alleles in both cases (S5B and S5C Fig). Similar levels of enrichment for the euchromatic allele were evident from 8 hours post transfection, substantially before the majority of mutations arise (Fig 5B), through to 96 hours. We conclude that heterochromatin impairs the kinetics of mutagenesis by inhibiting Cas9 occupancy (S5 Fig), and to a degree that depends on the level of intracellular Cas9 expression (Fig 4). However, target sites within heterochromatin ultimately reach similar frequencies of mutation upon sustained CRISPR exposure (Fig 5).

The repair of DSBs induced by Cas9-independent routes is thought to be influenced by the preexisting chromatin environment at the site of cleavage [18,20–22]. However, whether DNA accessibility and/or epigenetic modification of DNA and histone proteins can influence the outcome of CRISPR mutagenesis, particularly the frequency of InDels arising via NHEJ versus precise edits templated by exogenous nucleic acid donors (HDR), is not known. Imprinted genes provide an ideal system with which to address this question.

**Fig 6:**
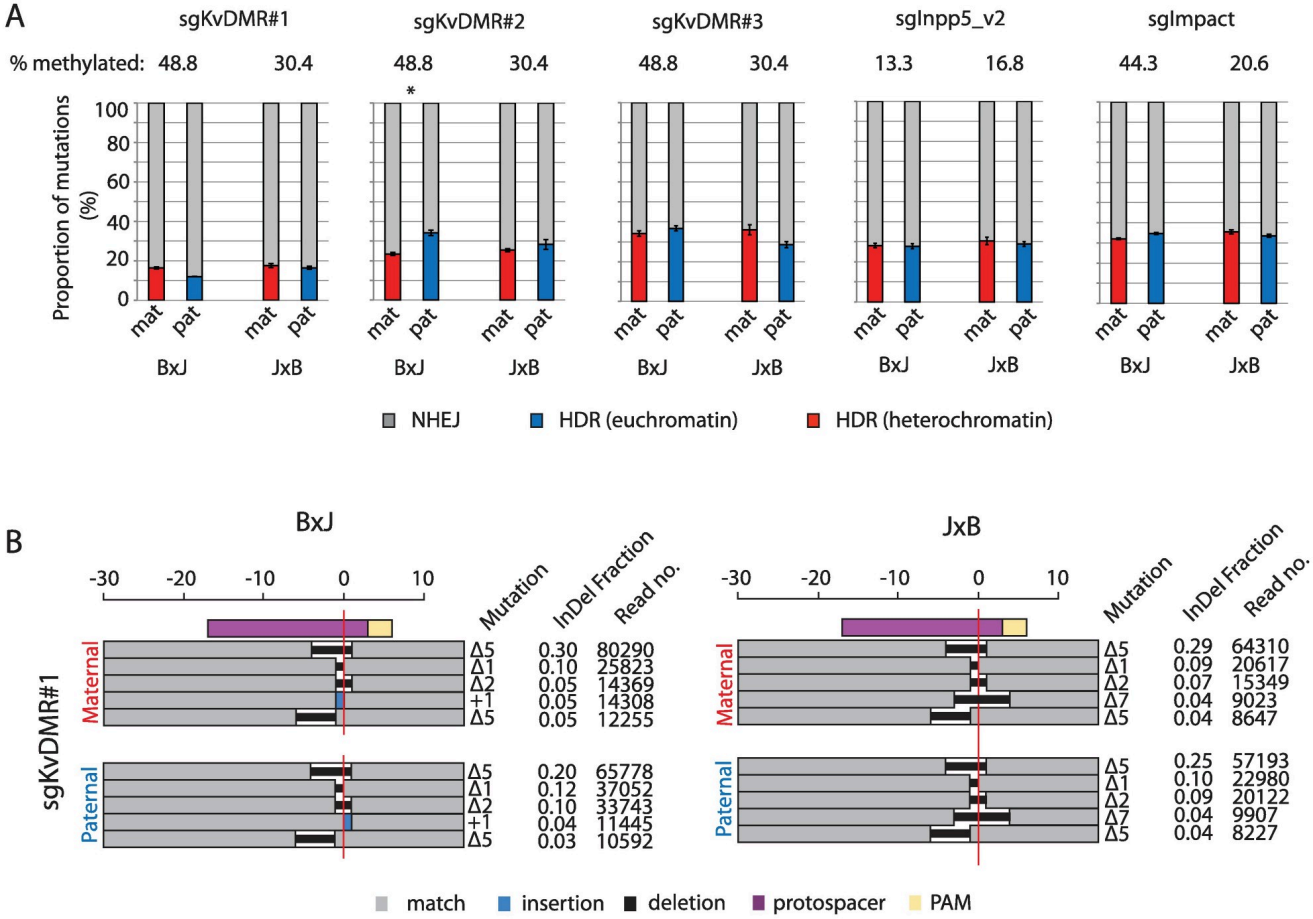
The ratio of InDels to precise sequence edits is unaffected by heterochromatin. (A) The relative frequency of mutations arising from NHEJ versus HDR in cells collected at 96 hours post transfection for five sgRNAs with target sites within imprinted heterochromatin (S1A, S2A and S3A Figs). The percentage of hypermethylated (>80%) strands in mock-transfected cells is indicated above the plot. Asterisk denotes Bonferroni-corrected *p* < 0.05 from paired *t* tests of difference between HDR frequencies on maternal versus paternal alleles. (B) The size and frequency of the top five most common InDels 96 hours following transfection with sgKVDMR#1 (S1A Fig), broken down by parental allele. The horizontal red line denotes the predicted cleavage site. Deletion sizes are depicted against the scale bar at the top, and for insertions, the number of inserted bases is indicated next to the blue rectangle. Note that different +1 nucleotide insertions were observed and are represented as blue rectangles on opposite sides of the cleavage site. The fraction of Indels was calculated as the number of reads corresponding to each specific mutation expressed as a proportion of all InDel-containing reads. Quantitative data underlying both panels are provided in S1 Data, and details of MiSeq libraries including SRA accessions are provided in S2 Data. HDR, homology-directed repair; InDel, Insertion or deletion; mat, maternal; NHEJ, nonhomologous end joining; PAM, protospacer adjacent motif; pat, paternal; sgRNA, single guide RNA; SRA, Sequence Read Archive.

For five sgRNAs targeting imprinted heterochromatin, mutational profiles were calculated separately from sequencing reads originating from maternal (repressed) versus paternal (active) alleles (Materials and methods). No consistent allelic biases were evident in the ratio of InDels versus HDR-derived edits at 4 days post transfection (Fig 6A), but the rate of HDR varied by up to 3-fold between loci. This suggests that DNA sequence features of the target and HDR template molecules [5,39] are more important than epigenetic properties in determining HDR efficiency. It is important to stress that our assay is designed to detect DNA sequence changes rather than DSB repair and cannot, in its current form, measure nonmutagenic repair that does not lead to genome edits (see Discussion). Nonetheless, the data suggest that the relative frequency of InDels versus HDR-derived edits, a key parameter in genome editing experiments, is not substantially affected by the preexisting state of local chromatin. We note that a recent study in *Drosophila* found that DSB repair kinetics and pathway choice were similar in euchromatin versus heterochromatin following I-SceI cleavage [40].

Next, we asked whether chromatin modifications influenced the spectrum and frequency of different InDel mutation classes produced via NHEJ. In line with a recent large-scale deep sequencing study of InDels induced by Cas9 in cancer cell lines [24], we found that each sgRNA produced its own characteristic InDel pattern, with the top five recurrent mutations comprising 30% to 60% of all sequencing reads in cells collected 96 hours following transfection (Fig 4D, S5 Fig). The same mutations recurred on maternal and paternal chromosomes (Fig 4D, S5 Fig) despite these allelic target sites starting in very different epigenetic states (Fig 1C and 1D, S1, S2 and S3 Figs). Thus, neither the relative frequency of InDels versus HDR edits nor the spectrum of different InDels is substantially affected by the presence of heterochromatin at a CRISPR target site.

## Discussion

In this study, we have used the classical epigenetic model system of genomic imprinting to determine the effect of chromatin context on CRISPR-Cas9 genome editing. This internally controlled approach allowed us to identify key experimental parameters (intracellular Cas9 expression level and duration of exposure) that determine the extent to which repressed chromatin impairs mutagenesis. Our findings are consistent with and extend those of previous studies in this area. The inhibitory effect of nucleosomes on Cas9 binding and cleavage is well established [7,8,11], and the sgRNAs used in this study targeted regions of allele-specific DNAse hypersensitivity (S1B, S2B and S3B Figs). On hypersensitive alleles, nucleosome–DNA interactions are less stable due to chromatin remodelling activities associated with RNA Polymerase II transcription [41]. It is also possible that DNA methylation can directly influence Cas9 binding and/or cleavage, and future studies will be required to address this possibility in vitro.

We propose that the dynamic nature of chromatin at these sites would provide more opportunities for Cas9 complexes to bind and cleave their targets per unit of time. Conversely, mutations accumulate more slowly in heterochromatin, where nucleosomes marked by H3K9me3 and H4K20me3 more effectively occlude Cas9 complexes during the target search [9]. Mutagenesis still occurs within heterochromatin, albeit at a slower rate, presumably due to residual nucleosome breathing [11] and remodelling associated with DNA replication. Future experiments should focus on post-mitotic cells, both to determine whether heterochromatin exerts a greater effect on mutagenesis in the absence of DNA replication as well as to remove the potentially confounding effect of cell cycle stage on DNA repair outcome [42].

Elevated concentrations of Cas9 increase the likelihood of mutation through mass action: an effect that we observed in both heterochromatin and euchromatin (Fig 4), and which caused mutagenesis to reach saturation more rapidly. Under conditions of low Cas9 expression and brief exposure in which no LOI occurred (Fig 1D), heterochromatin impeded mutagenesis by more than 6-fold (S4 Fig). In practical terms, our findings suggest that chromatin state is a particularly important consideration during procedures in which the level of Cas9 exposure is kept low. This would be relevant in a clinical setting, in which it is desirable to minimise exposure in order to avoid undesirable off-target mutations [43].

We also addressed, to our knowledge for the first time, whether local chromatin state influences the relative frequency of precise CRISPR-Cas9 genome edits (i.e., templated from exogenous single-stranded donor DNA) versus InDels. We found that this important aspect of genome editing was not significantly different between heterochromatin and euchromatin. This is somewhat surprising in light of prior reports that chromatin modifications influence DSB repair pathway choice in other contexts [20,21,44]. However, our study differs from these prior reports in several important ways. Firstly, we used Cas9 to introduce DSBs rather than restriction enzymes or ionising radiation. It is possible that localised chromatin remodelling events associated with Cas9 binding [16,17] are sufficient to overcome any prior differences in chromatin state at imprinted loci, which might otherwise influence the outcome of DNA repair. Secondly, it has been suggested that the repair of Cas9-derived DSBs could occur with unusually slow kinetics [45], possibly due to the presence of an R-loop proximal to the broken ends. Caution should therefore be exercised when generalizing findings from Cas9-induced breaks to those arising from other sources. Thirdly, our assay was designed to quantify genome editing rather than DSB repair and fails to report on nonmutagenic breaks repaired via NHEJ without end resection [46] or homologous recombination from the sister chromatid. Although our data show that the preexisting chromatin status does not affect the spectrum of mutations induced during genome editing, we emphasise that they do not directly assess the influence of chromatin on repair pathway choice.

In summary, we show that allele-specific epigenetic model systems such as genomic imprinting can provide new insights into mechanisms of genome editing in a physiological setting. Given the expanding range of synthetic DNA binding proteins now used in research, biotechnology, and medicine [47–51], this approach can provide further insights into their mode of interaction with chromatin in vivo. A strength of our assay is that it allows the influence of chromatin modifications to be assessed independently from the underlying DNA sequence. However, it is important to keep in mind that imprinted heterochromatin spans small genomic distances (approximately 2–10 kb) and is embedded within genomic euchromatin [26], similar to dispersed transposon sequences. Most genomic heterochromatin is assembled upon larger regions of clustered repeats such as pericentromeres and telomeres, in which the unusual DNA sequence properties are likely to influence target recognition and repair independently of chromatin status. Whether mechanisms of genome editing within imprinted heterochromatin reflect those in pericentromeric regions [52] therefore remains to be seen. In the future, it will be of interest to extend this study to assess other allele-specific chromatin states, such as transcribed versus nontranscribed imprinted gene bodies, and targets on the active versus inactive X chromosome.

## Materials and methods

### Ethics statement

Institutional Review Board approval for the usage of C57BL6/J and JF1 mice was obtained from the Reseaux Animalerie de Montpellier, Montpellier, France.

### Cell culture and transfection

Hybrid mESCs were derived in serum-free (2i) medium with LIF, Mek inhibitor PD0325901 (1 μM), and Gsk3 inhibitor CHIR99021 (3 μM) as described previously [53] and were maintained in ESGRO 1i medium (LIF and Gsk3 inhibitor; Millipore, SF001-500P). Unless otherwise indicated, all experiments were performed on cells at passages 5–12. A modal chromosome number of 40 was confirmed by counting metaphase chromosomes of cells at passage 11.

To derive high-passage cells with reduced CpG methylation at imprinted regions, B×J cells were cultured to passage 9 under 1i conditions before being switched into 2i medium for an additional 11 passages with daily media changes. From passages 20–22, ascorbic acid was added to the 2i media at a concentration of 100 μg/mL, then cells were moved back into ESGRO 1i medium for a single passage prior to transfection at passage 23.

Protospacer sequences were selected using the online tool hosted by the Broad Institute (https://portals.broadinstitute.org/gpp/public/analysis-tools/sgrna-design) within three loci previously described in the literature to exhibit allele-specific CpG methylation [54]. In all editing experiments, *Streptococcus pyogenes* Cas9 and sgRNA were both expressed from the same plasmid. pSpCas9(BB)-2A-GFP (PX458; Addgene plasmid #48138) and pSpCas9(BB)-2A-Puro (PX459; version 2.0; Addgene plasmid #62988) were gifts from Feng Zhang [35]. Plasmids were transfected together with a 150-nucleotide ssODN which served as a template for HDR. ssODNs introduced nucleotide substitutions that removed the NGG protospacer adjacent motif (PAM) to prevent further cleavage. For the experiments presented in Figs 4 and 5G and S4 Fig, sgRNA and Cas9-2A-eGFP were expressed from plasmid backbone pX458, whereas all other experiments used plasmid backbone pX459v2 [35]. Sequences of guides and donor oligonucleotides are listed in S3 Data. Transfections were performed in duplex, i.e., each transfection mix contained two separate plasmids encoding sgRNA and ssODNs to target two loci simultaneously. Experiments examining the effect of Cas9 expression level on mutagenesis (Figs 4 and 5G and S4 Fig) were the exception; here, plasmids were transfected individually.

Approximately 16 hours before transfection, 3 × 10^5^ cells were seeded in each well of a 6-well plate. Transfections were conducted using Lipofectamine 2000 (Invitrogen) according to the manufacturer’s protocol, with the following modification: transfection mix comprised a total of 3 μg plasmid and 150 ng oligonucleotide donor in 10 μL of P2000 reagent. Transfection efficiencies ranged from 15% to 50%. For all editing experiments that did not involve time points or Cas9-2A-eGFP selection, successfully transfected cells were selected in medium containing puromycin (1.6 μg/mL) 24 hours following transfection. Puromycin was washed out together with dead cells at 48 hours following transfection; then, genomic DNA was harvested from pooled cells at 96 hours. For the experiments in Figs 4 and 5G and S4 Fig, cells were FACS purified using the gating strategy shown in Fig 4B at 24 hours following transfection. Each sorted population was split 50:50, with half harvested immediately and the remainder after an additional 72 hours in culture. Transfected cells were not selected during any of the time course experiments presented in Fig 5. Sequences of guides and donor oligonucleotides are listed in S3 Data.

### Locus-specific amplification and MiSeq library preparation

DNA was isolated from edited cells using the DNeasy Blood and Tissue Kit (Qiagen) with RNAse treatment according to the manufacturer’s protocol. Each biological replicate used 50 ng of template DNA, corresponding to 8,333 diploid genomes. Adaptors and barcodes necessary for multiplexed high-throughput amplicon sequencing were added using a two-round PCR procedure. In the first round, locus-specific primers were designed to span regions encompassing both the editing site and an allelic SNP, which allowed the origin of each sequence read to be traced to the maternal or paternal allele. First-round primers contained 5′ extensions with a random hexamer, binding sites for Illumina sequencing primers, and binding sites for universal primers necessary for the second round of cycling. Edited loci were amplified for 25 cycles using High Fidelity Phusion Polymerase (NEB). PCR products were purified using AMPure beads (Beckman Coulter) according to the manufacturer’s instructions and eluted in 50 μL. A total of 10 μL of eluate was taken forward to a second round of PCR for eight cycles. The second round of PCR used universal primers that contained unique indices based on the i5 and i7 sequences from the Nextera library prep kit (Illumina). This enabled multiplexing of libraries on a single flow cell. Locus-specific and universal primer sequences are listed in S3 Data. Amplified products were purified using AMPure beads and eluted in 25 μL, and then concentration and product size were verified on an Agilent Bioanalyser. Libraries were pooled at equimolar ratio and run on an Illumina MiSeq to obtain 150 bp paired-end reads. Sequence data have been deposited at the Sequence Read Archive (SRA) under project accession PRJNA421037. Individual library details including read numbers are listed in S1 Data.

### Bisulphite sequencing

DNA was purified from unedited control cells harvested at equivalent passage number to edited populations (passage 6–12, unless otherwise stated) using the DNeasy Blood and Tissue Kit (Qiagen). A total of 0.5 μg of DNA was subjected to bisulphite conversion using the EZ DNA methylation kit (Zymo) according to the manufacturer’s instructions. Each converted sample was eluted in a 10 μL volume, of which 2 μL was used as a PCR template. The generation of libraries for Illumina sequencing proceeded as described above with the following modifications: the first round of PCR comprised 35 cycles rather than 25, and GoTaq Green (Promega) was used in place of Phusion Taq polymerase. A single library was generated for each locus.

### ChIP

The H3K9me3 ChIP-Seq track (GSM1000147) shown in Fig 1B and S1A Fig is from the ENCODE mESC line BRUCE4 (C57BL/6J strain), visualised using the UCSC genome browser on GRC37/mm9. All histone modification ChIP assays presented in Fig 1, S1, S2 and S3 Figs were performed on the hybrid mESC lines used for mutagenesis studies. H3K9me3 (07–442, batch 2664282) and H4K20me3 (07–643, batch 2586586) antibodies used in ChIP experiments were purchased from Millipore. Approximately 1 × 10^7^ cells were harvested at approximately 80% confluency, trypsinised, and washed in ice-cold phosphate buffered saline (PBS). Following centrifugation at 500 g, cells were resuspended in 1 mL of ice-cold NBA buffer (85 mM NaCl, 5.5% sucrose, 10 mM Tris-HCl [pH 7.5], 0.2 mM EDTA, 0.2 mM PMSF, 1 mM DTT, protease inhibitors). A total of 1mL of NBB buffer (NBA buffer with 0.1% NP-40) was added, and cells were incubated for 5 minutes on ice, then centrifuged at 1,000 g for 5 minutes at 4 °C. The pellet was resuspended in 200 μL of NBR buffer (85 mM NaCl, 5.5% sucrose, 10 mM Tris-HCl [pH 7.5], 3 mM MgCl_2_, 1.5 mM CaCl_2_, 0.2 mM PMSF, 1 mM DTT) and centrifuged for an additional 5 minutes at 4 °C, then resuspended in 600 μL NBR buffer. A total of 10 μLL of RNAseA (10 mg/mL) was added and incubated for 5 minutes at room temperature. A total of 40 Boehringer units of MNase (Sigma) were added, mixed by pipetting, and incubated at 20 °C for 10 minutes, with an additional mix by pipetting after 5 minutes. Digestion was stopped by adding 600 μL of MNase stop buffer (215 mM NaCl, 10 mM Tris-HCl [pH 8], 20 mM EDTA, 5.5% sucrose, 2% TritonX100, 0.2 mM PMSF, 1 mM DTT, 2x PMSF), and samples were stored at 4 °C overnight.

A total of 40 μL of protein A dynabeads (Invitrogen) were used per sample. After prewash in block solution (0.5% BSA in PBS), beads were mixed with 2.5 μg antibody in 1 mL block solution, incubated for 2 hours on a rotating wheel at 4 °C, and then washed in 200 μL block solution. Chromatin was centrifuged at 13,000 RPM for 5 minutes at 4 °C, and the supernatant was transferred to a fresh tube with 10% set aside for use as input. The amount of 1 mL of supernatant was added to the antibody bound beads together with 5 μL of BSA (5 mg/mL) before incubation at 4 °C for 3 hours on a rotating wheel.

Three washes with ChIP-W1 buffer (150 mM NaCl, 10 mM Tris-HCl [pH 8], 2 mM EDTA, 1% NP40, 1% sodium deoxycholate) were performed in 1 mL volume on a rotating wheel for 10 minutes at 4 °C, followed by 1 wash in TE Buffer at room temperature without rotation. After the last wash, beads were resuspended in 100 μL of elution solution (0.1 mM NaHCO_3_, 1% SDS), vortexed briefly, and incubated at 37 °C in a shaking thermomixer at 700 RPM. The pH was adjusted to pH 8 by adding 7 μL of 2M Tris-HCl (pH 6.8). Dynabeads were removed, and the remaining solution (and input samples) was treated with 20 μg of proteinase K for 1 hour at 55 °C. ChIP and input DNA were purified on Qiagen PCR purification columns.

For dCas9 ChIP, 1.8 × 10^6^ cells were seeded in each of 5 × 10 cm dishes 16 hours before transfection using Lipofectamine 2000 (Invitrogen). A total of 24 μg of plasmid was transfected per dish. The dCas9 backbone plasmid pX330A_dCas9-1x4 was a gift from Takashi Yamamoto (Addgene plasmid #63598) [55]. Cells were crosslinked in 1% formaldehyde at a density of 2 × 10^6^ per mL for 10 minutes, and then glycine (0.125 M final concentration) was added for an additional 10 minutes. A single wash in ice-cold PBS was followed by incubation in Farnham lysis buffer (5 mM PIPES [pH 8.0], 85 mM KCl, 0.5% NP-40, protease inhibitors), centrifugation, and storage of pellets at −80 °C. Upon thawing, a single wash in Farnham lysis buffer was followed by resuspension in RIPA buffer at 1 × 10^7^ cells per mL. Chromatin was sonicated to a fragment size of approximately 3 to 5 kb.

A total of 40 μL of protein G Dynabeads (Invitrogen) was used per sample. After prewash in block solution (0.5% BSA in PBS), beads were mixed with 5 ug antibody (Monoclonal ANTI-FLAG M2, F1804 Sigma) in 1 mL block solution per sample, incubated for 2 hours on a rotating wheel at 4 °C, and then washed in 200 μL block solution. The sonicated mixtures were spun at 13,000 RPM for 15 minutes at 4 °C to remove impurities. The amount of 1 mL from each sonicated mixture was used for the IP (900 μL to the beads with antibody and 90 μL maintained as 10% Input). IP samples were incubated at 4 °C for 18 hours on a rotating wheel.

A total of 5 × 1 mL washes with LiCl Wash Buffer (100 mM Tris [pH 7.5], 500 mM LiCl, 1% NP-40, 0.5% sodium deoxycholate) were performed on a rotating wheel for 5 minutes at 4 °C, followed by 1 wash in TE Buffer at room temperature without rotation. After the last wash, beads were resuspended in 200 μL of elution solution (0.1 mM NaHCO_3_, 1% SDS), vortexed briefly, and incubated for 1 hour at 65 °C in a shaking thermomixer at 1,000 RPM. Dynabeads were removed, and the remaining solution (and input samples) was treated with 20 μg of proteinase K for 30 minutes at 37 °C. A total of 40 μg proteinase K was added to each sample, and these were incubated for 4 to 5 hours at 65 °C to complete reversal of cross links. ChIP and input DNA were purified on Qiagen PCR purification columns.

For relative quantification of ChIP DNA by real-time qPCR, DNA isolated from 10% of total MNase digested native chromatin was used to generate a standard curve (5-fold dilutions, from 10% to 0.08% total input) for IP samples. qPCR was performed in triplicate using SYBR Select mastermix (Applied Biosystems) on a LightCycler 480 II (Roche) with thermal cycling as follows: Initial Cycle 50 °C for 2 minutes and 95 °C for 2 minutes, and then 40 cycles of 95 °C for 15 seconds, 60 °C for 50 seconds, and 60 °C for 10 seconds with a single acquisition. A total of 0.5 μL input or ChIP DNA was used in a total reaction volume of 20 μL. For allele-specific enrichment analysis, regions spanning an allelic SNP were amplified using GoTaq (Promega), and amplicons were purified using AMPure beads and then subjected to Sanger sequencing. Primer sequences are listed in S3 Data.

### DNase-I accessibility assay

DNAse-I digestion was performed using a published protocol [56] with the following modifications. A total of 20 × 10^6^ cells were trypsinised and resuspended in 5 mL buffer A (15 mM Tris-HCl [pH 7.6], 60 mM KCl, 15 mM NaCl, 1 mM EDTA, 0.5 mM EGTA, 0.5 mM spermidine, 0.15 mM spermine). Cells were lysed in the presence of 0.5% (v/v) NP40, and nuclei were collected by centrifugation (2000 g/5 minutes) and resuspended in 1 mL digestion buffer (buffer A supplemented with 3 mM CaCl_2_, 75 mM NaCl). Digestions were carried out at 37 °C with 0–60 units of DNase-I (Sigma) per 100 μL nuclei, for 5 minutes before the reaction was stopped by the addition of an equal volume of stop buffer (0.1 M NaCl, 0.1% [w/v] SDS, 50 mM Tris-HCl [pH 8.0], 100 mM EDTA). The samples were treated with 2 μg proteinase K at 55 °C overnight, and DNA was recovered after extraction with phenol/chloroform and precipitation in ethanol. The DNA was then resuspended in TE buffer (10 mM Tris-HCl [pH 8.0], 1 mM EDTA), and concentration was measured using fluorometric quantitation (Qubit). Digested DNA was amplified for 30 cycles across regions containing an allelic SNP. Amplicons were purified using AMPure beads and then subjected to Sanger sequencing across regions of 300 to 600 bp spanning an allelic polymorphism. Primer sequences are listed in S3 Data.

### Analysis of high-throughput sequencing data

#### All samples

Reads were de-multiplexed and duplicate read pairs removed by FastUniq version 1.1 [57], and adaptors were trimmed with TrimGalore version 0.4.1 (https://www.bioinformatics.babraham.ac.uk/projects/trim_galore/).

#### Genomic sequencing of edited samples

Trimmed and de-duplicated read pairs were aligned to mouse genome build GRCm38 using BWA version 0.7.12 [58]. Read pairs were extracted by the expected genomic region for each experiment and were assigned to the C57BL/6J or JF1 chromosome based on nucleotide identity at known polymorphic SNPs (http://molossinus.lab.nig.ac.jp/msmdb/index.jsp). Read pairs containing mutations originating from HDR were identified based on the expected sequence changes introduced from the oligonucleotide donors (S3 Data), whereas read pairs containing InDels within 10 bp of the cleavage site were identified as originating from fragments that had undergone NHEJ. Read pairs with evidence of neither were labelled as wild type. Indel length and type (insertion or deletion) were extracted from the NHEJ read pairs via a custom Perl script.

#### Bisulphite sequencing of unedited samples

Trimmed and de-duplicated read pairs were aligned to the bisulphite conversion indexed mouse genome build GRCm38 using Bismark version 0.16.3 [59] with Bowtie version 2.2.6 [60]. Read pairs that did not align were then separated, and each end of the pair was aligned as single end reads. The three resulting alignments were merged. Read pairs were extracted by the expected genomic region for each experiment. The number of methylated CpGs in each read pair was counted using a custom Perl script examining the XM tag for each read in the relevant BAM file. Because not all sequencing amplicons contained an informative SNP to distinguish parental alleles, we report total methylation levels across both alleles and do not distinguish maternal from paternal strands. All sequencing data have been deposited in the SRA under project accession PRJNA421037.

## Supporting information

S1 Fig Allele-specific chromatin states at the imprinted *KvDMR* locus. (A) UCSC screen drop showing the *KvDMR* locus, including the transcriptional start site for the *Kcnq1ot1* noncoding RNA, which is active from the paternal allele. H3K9me3 ChIP and DNase-I–seq data from mESCs are available through EncODE (ENCSR000CFZ, GSM1014187). Positions of sgRNA target sites and PCR amplicons used during the analysis are indicated. B. Allele-specific DNase-I sensitivity of regions indicated in panel A. Note that Target 2 is within an annotated DNase-I hypersensitive site, whereas Target 1 is not. mESC nuclei were subjected to digestion with increasing concentrations of DNase-I for 5 minutes at 37 °C, before DNA extraction and Sanger sequencing across SNPs to reveal allele-specific differences in digestion at the regions indicated in panel A. (C) Native ChIP enrichment for H3K9me3 and H4K20me3 marks at regions corresponding to sgRNA target site 1, and 2&3 (amplicons indicated in panel A). Enrichments are expressed relative to input, and error bars represent SD of three technical replicates. qPCR primers spanning Intracisternal A particle (IAP) retrotransposons and the actb promoter serve as positive and negative controls, respectively. D. Allele-specific enrichment in ChIP DNA for the Target 1 region shown in panel A determined by RFLP analysis. The data are representative of two biological replicates for each mESC line. Quantitative data underlying panel C are provided in S1 Data, and details of MiSeq libraries including SRA accessions are provided in S2 Data. SRA, Sequence Read Archive.
(TIF)

S2 Fig Allele-specific chromatin states at the imprinted *Impact* locus. (A) UCSC screen drop showing the transcriptional start site for the *Impact* gene, which is active from the paternal allele. H3K9me3 ChIP and DNase-I–seq data from mESCs are available through EncODE (ENCSR000CFZ, GSM1014187). Positions of the sgRNA target site and PCR amplicons used during the analysis are indicated. (B) Allele-specific DNase-I sensitivity for a region spanning the target site, as indicated in panel A. (C) ChIP enrichment for H3K9me3 and H4K20me3 marks at the *Impact* sgRNA target site. Enrichments are presented in the same manner as S1C Fig. (D) Allele-specific enrichment of ChIP DNA at the *Impact* sgRNA target site determined by Sanger sequencing from ChIP DNA across an allelic SNP. ChIP experiments are representative of two biological replicates for each mESC line. (E) CpG methylation at the *Impact* promoter presented as described for Fig 1D. The black dashed line indicates the expected level of methylation across all alleles when imprinting is completely maintained. (F) Allele-specific mutation analysis from experiments using sgImpact in cells collected 96 hours post transfection. Data are presented as described in Fig 2. Error bars depict SD, *n* = 3 biological replicates. Quantitative data underlying panels C, E, and F are provided in S1 Data, and details of MiSeq libraries including SRA accessions are provided in S2 Data. SRA, Sequence Read Archive.
(TIF)

S3 Fig Allele-specific chromatin states at the imprinted *Inpp5f_v2* locus. (A) UCSC screen drop showing the transcriptional start site for the *Inpp5f_v2* transcript, which initiates from the paternal allele. H3K9me3 ChIP and DNase-I–seq data from mouse ESCs available through EncODE (ENCSR000CFZ, GSM1014187). Positions of the sgRNA target site and PCR amplicons used during the analysis are indicated. (B) Allele-specific DNase-I sensitivity for a PCR amplicon spanning the *Inpp5f_v2* sgRNA target site, as described in S1B Fig. ND = not done due to poor PCR amplification in these samples. (C) ChIP enrichment for H3K9me3 and H4K20me3 marks at the *Inpp5f_v2* sgRNA target site. Enrichments are presented in the same manner as S1C Fig. (D) Allele-specific enrichment in ChIP experiments at the *Inpp5f_v2* sgRNA target site determined by RFLP analysis of PCR products amplified from ChIP DNA. ChIP experiments are representative of two biological replicates for each mESC line. (E) CpG methylation at the *Inpp5f_v2* promoter presented as described for Fig 1D. Methylation levels were measured separately following each transfection; the data shown here are representative. The black dashed line indicates the expected level of methylation across all alleles when imprinting is completely maintained. Note the partial LOI that is evident in panels B, D, and E, particularly in the B×J mESC line. Quantitative data underlying panels C and E are provided in S1 Data, and details of MiSeq libraries including SRA accessions are provided in S2 Data. SRA, Sequence Read Archive.
(TIF)

S4 Fig Heterochromatin impedes mutagenesis in a Cas9-concentration–dependent manner. (A) B×J cells from the transfection shown in Fig 2A were FACS purified according to the gating scheme shown. Note that this panel depicts the same data shown in panel 2A. (B) Allele-specific mutation analysis within cell populations expressing different levels of Cas9, as shown in panel A, FACS purified 24 hours post transfection and then subjected to allele-specific mutation analysis immediately, without further time in culture. Insufficient J×B cells were obtained following FACS to assess mutagenesis after 24 hours. (C) Stacked histograms show the allelic mutation bias in each experimental replicate. Error bars represent SD of three biological replicates. A one-way ANOVA was conducted using the fold-difference between mutation frequencies on maternal versus paternal alleles to determine whether this was affected by Cas9 expression level. Significant effects were found (*p* < 0.05). Asterisks denote *p*-values for Tukey’s HSD test on the specified pairwise comparisons. *p < 0.05. Quantitative data underlying panels B and C are provided in S1 Data, and details of MiSeq libraries including SRA accessions are provided in S2 Data. SRA, Sequence Read Archive.
(TIF)

S5 Fig Enhanced Cas9 occupancy in euchromatin compared to heterochromatin. (A) Schematic depicting the experimental workflow for Cas9 ChIP experiments. (B, C) Stacked histograms show ChIP enrichment for dCas9-3xFLAG at regions spanning the sgKvDMR#1 (panel B) and sgImpact (panel C), expressed relative to input DNA. Overall enrichments were determined by qPCR, and then separate PCR amplicons were subjected to amplicon deep sequencing in order to determine the ratio of products from maternal (red) to paternal (blue) alleles. Each time series was performed once in B×J cells only. Error bars represent SD of overall recovery from technical triplicate qPCR reactions. Quantitative data underlying panel B are provided in S1 Data, and details of MiSeq libraries including SRA accessions are provided in S2 Data. SRA, Sequence Read Archive.
(TIF)

S6 Fig The same InDel classes recur in heterochromatin and euchromatin 96 hours post transfection. The size and frequency of the top five most common InDels (broken down by parental allele) produced by four different sgRNAs targeting imprinted heterochromatin. Edited genomic DNA was extracted 4 days following transfection with sgRNAs targeting the KvDMR (panels A and B), *Impact* (panel C), and Inpp5f_v2 (panel D) imprinted loci in B×J (left) and J×B (right) cells. Deletion sizes are depicted against the scale bar at the top of each panel, and the number of inserted bases is indicated next to the blue rectangle. Note that any of four possible nucleotides can theoretically be inserted; therefore, more than one +1 insertion was observed in some instances. The horizontal red line denotes the predicted cleavage site, and the colour key for all panels is situated at the bottom left of the figure. The fraction of Indels was calculated as the number of reads corresponding to each specific mutation class, expressed as a proportion of all InDel-containing reads. The fraction of hypermethylated strands in mock-transfected cells is indicated below each plot. Details of MiSeq libraries including SRA accessions are provided in S2 Data. SRA, Sequence Read Archive.
(TIF)

S1 Data Quantitative data underlying summary figures. (XLSX)

S2 Data Details of Illumina sequencing libraries used in this study including SRA sample accessions. SRA, Sequence Read Archive.
(XLSX)

S3 Data Details of oligonucleotides used in this study. (XLSX)

## Abbreviations

Cas9: CRISPR-associated protein 9
ChIP: chromatin immunoprecipitation
CRISPR: clustered regularly interspaced palindromic repeats
crRNA: CRISPR RNA
dCas9: catalytically dead Cas9
DSB: double strand break
eGFP: enhanced green fluorescent protein
FACS: fluorescence-activated cell sorting
HDR: homology-directed repair
HSD: honest significant difference
InDel: Insertion or deletion
LOI: loss of imprinting
mESC: mouse embryonic stem cell
NHEJ: nonhomologous end joining
PAM: protospacer adjacent motif
PBS: phosphate buffered saline
RFP: red fluorescent protein
sgRNA: single guide RNA
SNP: single nucleotide polymorphism
SRA: Sequence Read Archive
ssODN: single-stranded oligodeoxynucleotide
