# Correction: Heterochromatin delays CRISPR-Cas9 mutagenesis but does not influence the outcome of mutagenic DNA repair

**DOI:** 10.1371/journal.pbio.3000160

**Published:** 2019-02-21

**Authors:** The PLOS Biology Staff

There is an error in the second sentence of the Author Summary. The correct sentence is: Imprinting has served as a model system to understand the mechanisms through which chromatin modifications can influence transcriptional regulation; comparisons between active and repressed alleles in the same cell nucleus provide an internal control for the effects of DNA sequence and exposure to diffusible regulators.
