# Supplementary figures and images for: Heterochromatin delays CRISPR-Cas9 mutagenesis but does not influence the outcome of mutagenic DNA repair
Source: PLoS Biol. 2018 Dec 12;16(12):e2005595. doi: 10.1371/journal.pbio.2005595 (PMC6306241; doi:10.1371/journal.pbio.2005595)

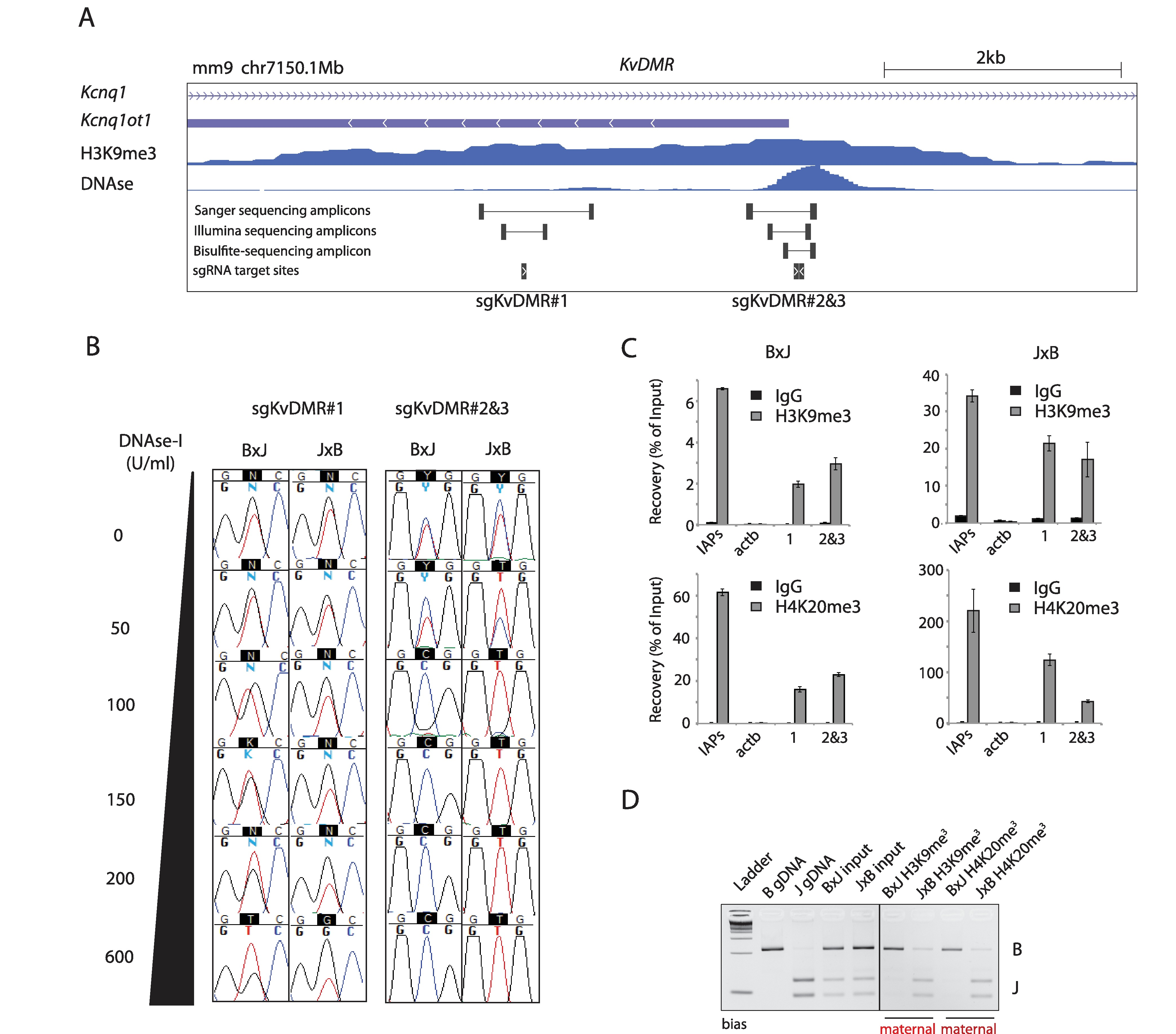

Supplement: S1 Fig — (A) UCSC screen drop showing the KvDMR locus, including the transcriptional start site for the Kcnq1ot1 noncoding RNA, which is active from the paternal allele. H3K9me3 ChIP and DNase-I–seq data from mESCs are available through EncODE (ENCSR000CFZ, GSM1014187). Positions of sgRNA target sites and PCR amplicons used during the analysis are indicated. B. Allele-specific DNase-I sensitivity of regions indicated in panel A. Note that Target 2 is within an annotated DNase-I hypersensitive site, whereas Target 1 is not. mESC nuclei were subjected to digestion with increasing concentrations of DNase-I for 5 minutes at 37 °C, before DNA extraction and Sanger sequencing across SNPs to reveal allele-specific differences in digestion at the regions indicated in panel A. (C) Native ChIP enrichment for H3K9me3 and H4K20me3 marks at regions corresponding to sgRNA target site 1, and 2&3 (amplicons indicated in panel A). Enrichments are expressed relative to input, and error bars represent SD of three technical replicates. qPCR primers spanning Intracisternal A particle (IAP) retrotransposons and the actb promoter serve as positive and negative controls, respectively. D. Allele-specific enrichment in ChIP DNA for the Target 1 region shown in panel A determined by RFLP analysis. The data are representative of two biological replicates for each mESC line. Quantitative data underlying panel C are provided in S1 Data, and details of MiSeq libraries including SRA accessions are provided in S2 Data. SRA, Sequence Read Archive. (TIF) [file pbio.2005595.s001.tif]

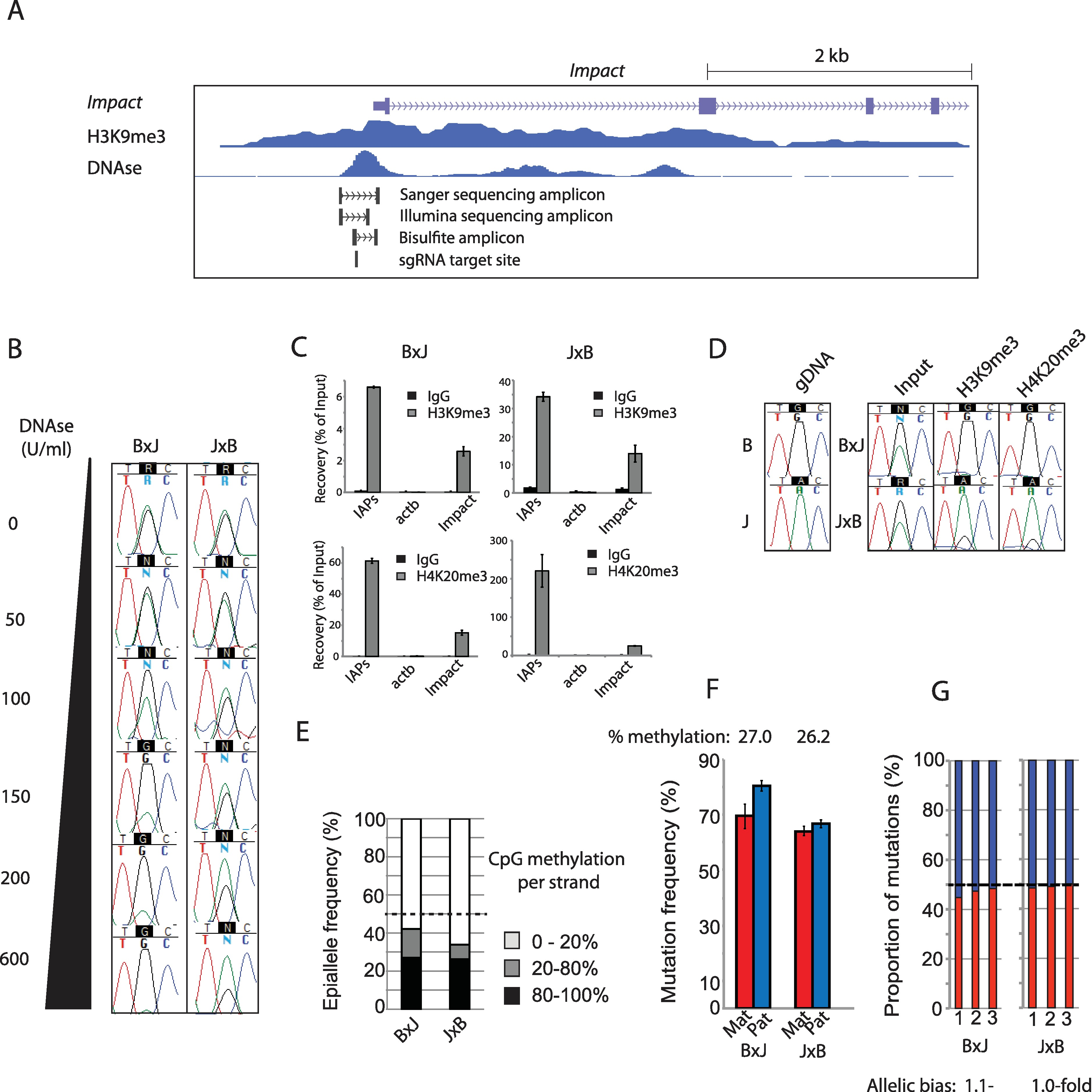

Supplement: S2 Fig — (A) UCSC screen drop showing the transcriptional start site for the Impact gene, which is active from the paternal allele. H3K9me3 ChIP and DNase-I–seq data from mESCs are available through EncODE (ENCSR000CFZ, GSM1014187). Positions of the sgRNA target site and PCR amplicons used during the analysis are indicated. (B) Allele-specific DNase-I sensitivity for a region spanning the target site, as indicated in panel A. (C) ChIP enrichment for H3K9me3 and H4K20me3 marks at the Impact sgRNA target site. Enrichments are presented in the same manner as S1C Fig. (D) Allele-specific enrichment of ChIP DNA at the Impact sgRNA target site determined by Sanger sequencing from ChIP DNA across an allelic SNP. ChIP experiments are representative of two biological replicates for each mESC line. (E) CpG methylation at the Impact promoter presented as described for Fig 1D. The black dashed line indicates the expected level of methylation across all alleles when imprinting is completely maintained. (F) Allele-specific mutation analysis from experiments using sgImpact in cells collected 96 hours post transfection. Data are presented as described in Fig 2. Error bars depict SD, n = 3 biological replicates. Quantitative data underlying panels C, E, and F are provided in S1 Data, and details of MiSeq libraries including SRA accessions are provided in S2 Data. SRA, Sequence Read Archive. (TIF) [file pbio.2005595.s002.tif]

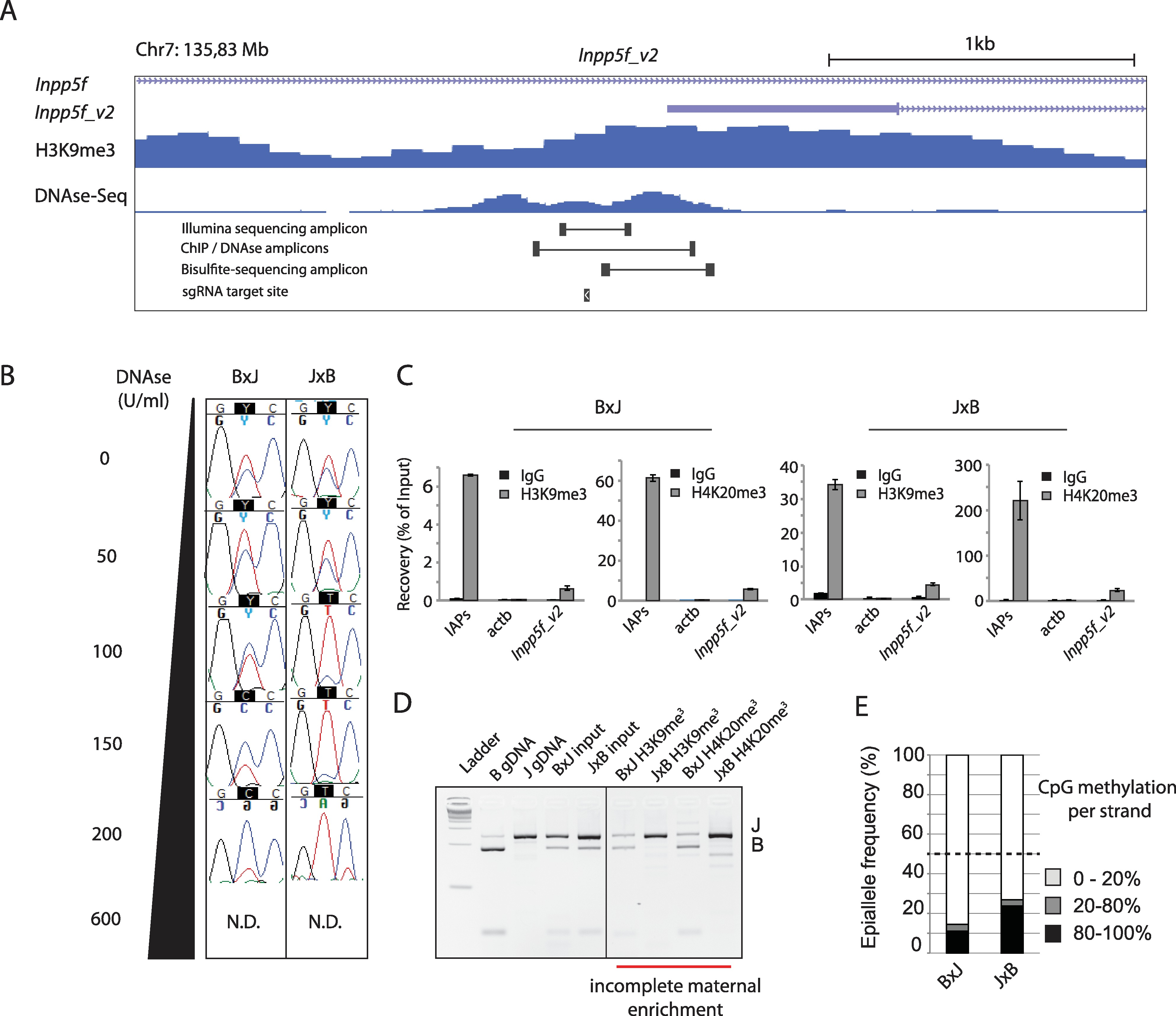

Supplement: S3 Fig — (A) UCSC screen drop showing the transcriptional start site for the Inpp5f_v2 transcript, which initiates from the paternal allele. H3K9me3 ChIP and DNase-I–seq data from mouse ESCs available through EncODE (ENCSR000CFZ, GSM1014187). Positions of the sgRNA target site and PCR amplicons used during the analysis are indicated. (B) Allele-specific DNase-I sensitivity for a PCR amplicon spanning the Inpp5f_v2 sgRNA target site, as described in S1B Fig. ND = not done due to poor PCR amplification in these samples. (C) ChIP enrichment for H3K9me3 and H4K20me3 marks at the Inpp5f_v2 sgRNA target site. Enrichments are presented in the same manner as S1C Fig. (D) Allele-specific enrichment in ChIP experiments at the Inpp5f_v2 sgRNA target site determined by RFLP analysis of PCR products amplified from ChIP DNA. ChIP experiments are representative of two biological replicates for each mESC line. (E) CpG methylation at the Inpp5f_v2 promoter presented as described for Fig 1D. Methylation levels were measured separately following each transfection; the data shown here are representative. The black dashed line indicates the expected level of methylation across all alleles when imprinting is completely maintained. Note the partial LOI that is evident in panels B, D, and E, particularly in the B×J mESC line. Quantitative data underlying panels C and E are provided in S1 Data, and details of MiSeq libraries including SRA accessions are provided in S2 Data. SRA, Sequence Read Archive. (TIF) [file pbio.2005595.s003.tif]

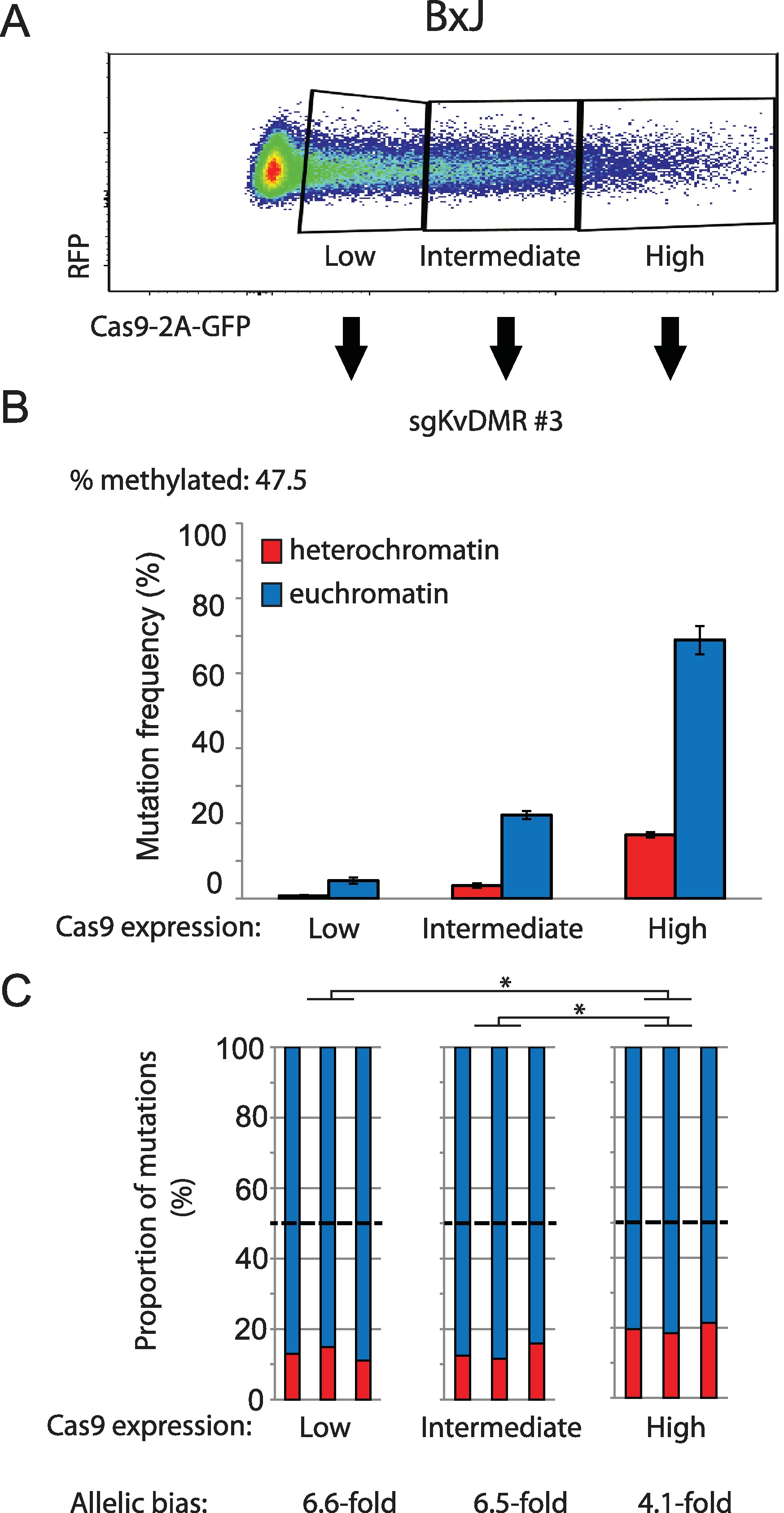

Supplement: S4 Fig — (A) B×J cells from the transfection shown in Fig 2A were FACS purified according to the gating scheme shown. Note that this panel depicts the same data shown in panel 2A. (B) Allele-specific mutation analysis within cell populations expressing different levels of Cas9, as shown in panel A, FACS purified 24 hours post transfection and then subjected to allele-specific mutation analysis immediately, without further time in culture. Insufficient J×B cells were obtained following FACS to assess mutagenesis after 24 hours. (C) Stacked histograms show the allelic mutation bias in each experimental replicate. Error bars represent SD of three biological replicates. A one-way ANOVA was conducted using the fold-difference between mutation frequencies on maternal versus paternal alleles to determine whether this was affected by Cas9 expression level. Significant effects were found (p < 0.05). Asterisks denote p-values for Tukey’s HSD test on the specified pairwise comparisons. *p < 0.05. Quantitative data underlying panels B and C are provided in S1 Data, and details of MiSeq libraries including SRA accessions are provided in S2 Data. SRA, Sequence Read Archive. (TIF) [file pbio.2005595.s004.tif]

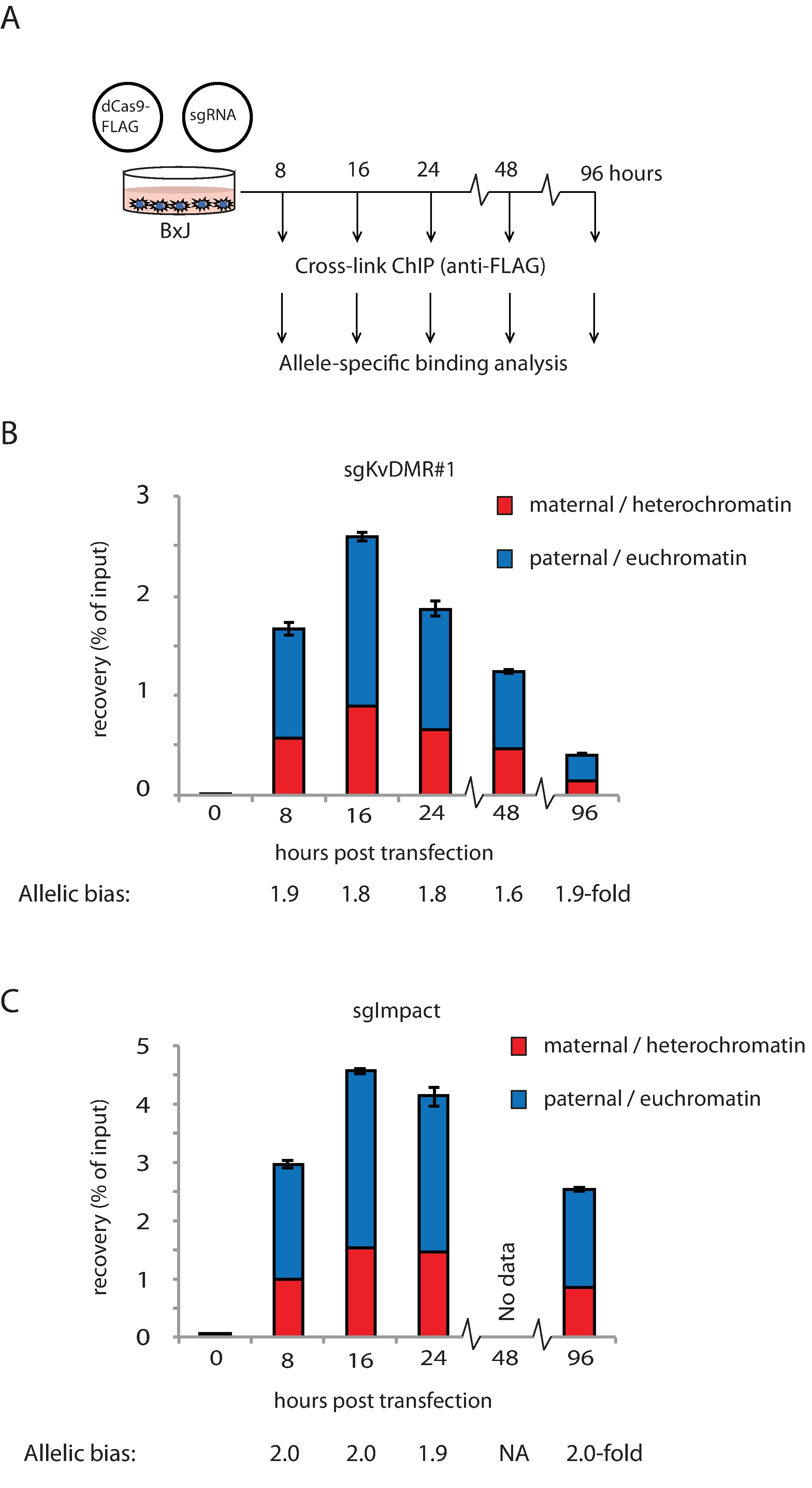

Supplement: S5 Fig — (A) Schematic depicting the experimental workflow for Cas9 ChIP experiments. (B, C) Stacked histograms show ChIP enrichment for dCas9-3xFLAG at regions spanning the sgKvDMR#1 (panel B) and sgImpact (panel C), expressed relative to input DNA. Overall enrichments were determined by qPCR, and then separate PCR amplicons were subjected to amplicon deep sequencing in order to determine the ratio of products from maternal (red) to paternal (blue) alleles. Each time series was performed once in B×J cells only. Error bars represent SD of overall recovery from technical triplicate qPCR reactions. Quantitative data underlying panel B are provided in S1 Data, and details of MiSeq libraries including SRA accessions are provided in S2 Data. SRA, Sequence Read Archive. (TIF) [file pbio.2005595.s005.tif]

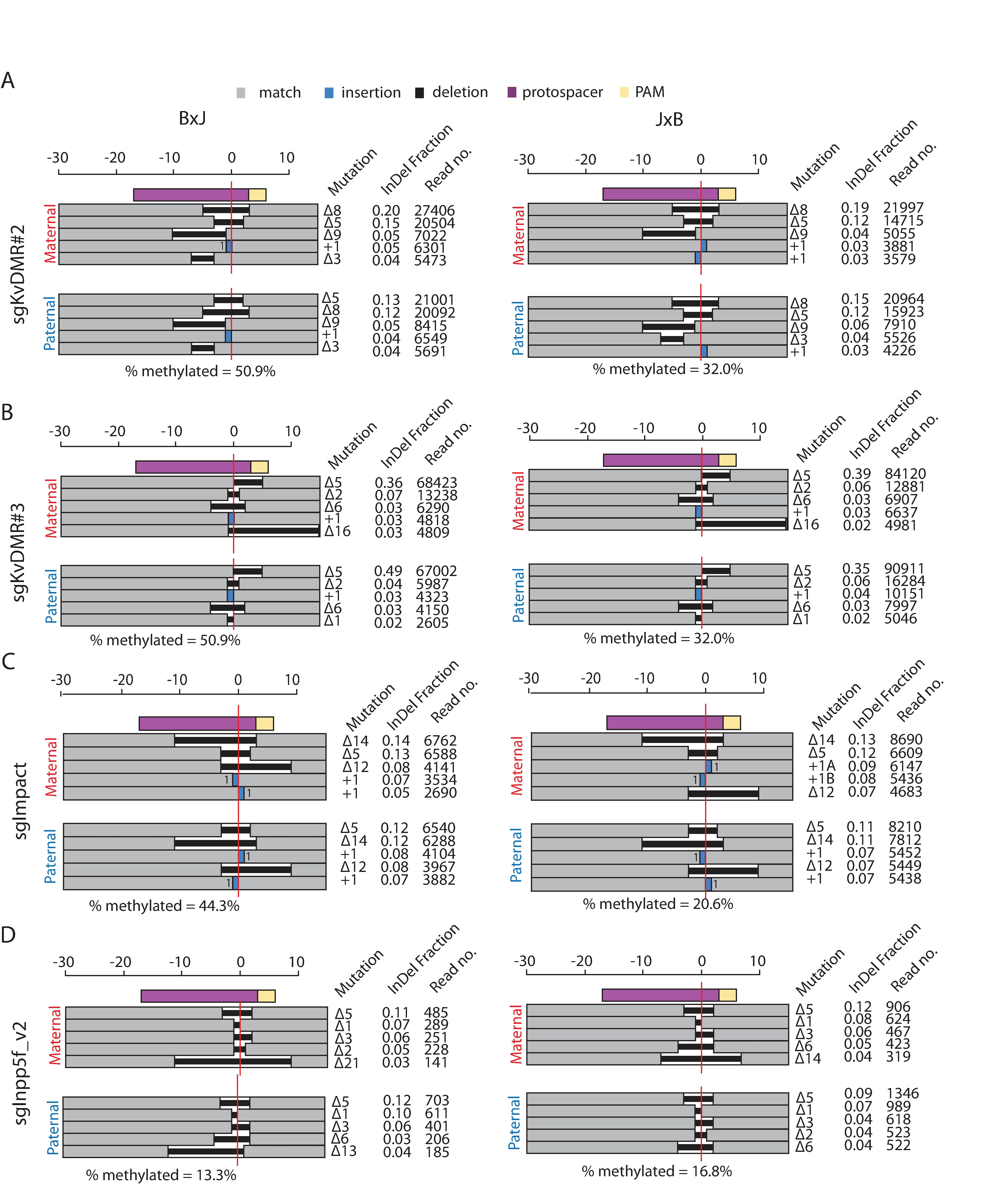

Supplement: S6 Fig — The size and frequency of the top five most common InDels (broken down by parental allele) produced by four different sgRNAs targeting imprinted heterochromatin. Edited genomic DNA was extracted 4 days following transfection with sgRNAs targeting the KvDMR (panels A and B), Impact (panel C), and Inpp5f_v2 (panel D) imprinted loci in B×J (left) and J×B (right) cells. Deletion sizes are depicted against the scale bar at the top of each panel, and the number of inserted bases is indicated next to the blue rectangle. Note that any of four possible nucleotides can theoretically be inserted; therefore, more than one +1 insertion was observed in some instances. The horizontal red line denotes the predicted cleavage site, and the colour key for all panels is situated at the bottom left of the figure. The fraction of Indels was calculated as the number of reads corresponding to each specific mutation class, expressed as a proportion of all InDel-containing reads. The fraction of hypermethylated strands in mock-transfected cells is indicated below each plot. Details of MiSeq libraries including SRA accessions are provided in S2 Data. SRA, Sequence Read Archive. (TIF) [file pbio.2005595.s006.tif]
